# The disparate impacts of college admissions policies on Asian American applicants

**DOI:** 10.1038/s41598-024-55119-0

**Published:** 2024-02-23

**Authors:** Joshua Grossman, Sabina Tomkins, Lindsay Page, Sharad Goel

**Affiliations:** 1 Department of Management Science and Engineering, Stanford University, Stanford, 94305 USA; 2 School of Information, University of Michigan, Ann Arbor, 48109 USA; 3 Education Department, Brown University, Providence, 02912 USA; 4 Kennedy School of Government, Harvard University, Cambridge, 02138 USA

**Keywords:** Computer science, Statistics, Computer science, Statistics

## Abstract

There is debate over whether Asian American students face additional barriers, relative to white students, when applying to selective colleges. Here we present the results from analyzing 685,709 applications submitted over five application cycles to 11 highly selective colleges (the “Ivy-11”). We estimate that Asian American applicants had 28% lower odds of ultimately attending an Ivy-11 school than white applicants with similar academic and extracurricular qualifications. The gap was particularly pronounced for students of South Asian descent (49% lower odds). Given the high yield rates and competitive financial aid policies of the schools we consider, the disparity in attendance rates is likely driven, at least in part, by admissions decisions. In particular, we offer evidence that this pattern stems from two factors. First, many selective colleges give preference to the children of alumni in admissions. We find that white applicants were substantially more likely to have such legacy status than Asian applicants. Second, we identify geographic disparities potentially reflective of admissions policies that disadvantage students from certain regions of the United States. We hope these results inform discussions on equity in higher education.

## Introduction

Over the last several decades, questions have been raised over whether selective colleges in the U.S. discriminate against Asian American applicants in admissions decisions^1–9^. In the 1980s, Brown and Stanford formed committees to audit their own admissions policies and practices^1,2^. Brown found evidence of discrimination in its admissions process; Stanford did not find clear evidence of bias, but could not fully explain its lower acceptance rates of Asian American applicants relative to white students. A 1990 report by the U.S. Department of Education’s Office of Civil Rights (OCR) investigated allegations that Harvard capped the number of Asian American students it admitted^1^. OCR found no evidence of an Asian quota, but concluded that Asian American applicants were less likely to be admitted than white students with similar academic qualifications. OCR further found that this disparity largely disappeared once recruited athletes and the children of alumni (“legacies”) were excluded from its analysis, suggesting the gap in acceptance rates was driven by Harvard’s stated preference for admitting students from these two groups^6,10,11^. Most recently, in a 2023 decision, the Supreme Court ruled that Harvard engaged in unconstitutional racial balancing, holding the Asian American share of admitted students to approximately 20%—though Harvard denied doing so. In the more than 30 years since the OCR investigation, there have been limited third-party, applicant-level empirical analyses of potential discrimination in college admissions decisions against Asian American applicants. Over this time span, both the demographics of the United States and the educational landscape have changed substantially. Asian American representation among K-12 public school students has more than doubled, increasing from 3% in 1993 to 7% in 2020^12^, and the overall admission rate to Harvard has dropped from 18% in 1990 to 5% in 2020^13,14^. These changes suggest a need to reexamine college admissions policies for potential disparate impacts on Asian American applicants.

Here we analyze 685,709 first-year college applications submitted by 292,795 Asian American and white students to the “Ivy-11”, an 11-college subset of 13 highly selective colleges often included among the “Ivy-Plus”. While there is not an agreed-upon definition of “Ivy-Plus”, the “Ivy-Plus” set of colleges most often includes all eight Ivy League colleges (Brown University, Columbia University, Cornell University, Dartmouth College, Harvard University, the University of Pennsylvania, Princeton University, and Yale University) along with one or more of five other institutions: the Massachusetts Institute of Technology, Duke University, the University of Chicago, Stanford University, and Northwestern University^15,16^. Identifying potential disparate impacts of “Ivy-Plus” admissions policies is of particular importance, as alumni of “Ivy-Plus” schools are disproportionately represented in positions of power^11^. For students admitted in the 2018–2019 admissions cycle, both these 13 “Ivy-Plus” colleges and the “Ivy-11” colleges we consider have yield rates between 54 and 82%, and acceptance rates between 4.2 and 10.6%^17,18^. The yield rate of a college is the probability an admitted student ultimately enrolls.

To respect the wishes of our data provider, we do not reveal the precise set of “Ivy-Plus” institutions included in the Ivy-11. All of the applications we consider were submitted via a national postsecondary application platform over five application cycles, from the 2015–2016 cycle to the 2019–2020 cycle. Each of the institutions we consider receives the majority of first-year applications from students applying via the application platform (Table S1). We exclude students who attended a high school outside of the United States or who reported primary citizenship outside of the United States. We further exclude students who we infer to be recruited athletes.

Given the complex patterns of immigration and marked heterogeneity in experiences across subgroups, we disaggregate our analysis by three regions of origin self-reported by the Asian American applicants in our dataset: South Asia, East Asia, and Southeast Asia. 3% of Asian applicants report multiple regions of origin. In these cases, we randomly assign one of the reported regions. 2% of Asian applicants do not select a region of origin. These students are excluded from the analysis.

A key limitation of our analysis is that we focus on whether applicants to Ivy-11 schools ultimately *attended* an Ivy-11 school, rather than whether they were *admitted* to an Ivy-11 school, as we do not have access to admissions decisions. However, given the competitive financial aid packages and high yield rates of Ivy-11 colleges, it seems likely that the disparities in enrollment that we identify are at least partially driven by disparities in admissions decisions—a point we return to in the Discussion. To preserve confidentiality, we focus on broader patterns rather than on individual institutions, and we report aggregate results across the combined set of Ivy-11 colleges.

We start by measuring disparities in Ivy-11 attendance after adjusting for academic and extracurricular accomplishment—traditional markers of “merit” in higher education. We estimate that South Asian applicants to Ivy-11 schools had 49% lower odds of attending an Ivy-11 school than white applicants with comparable test scores, high school grade-point averages, and extracurricular participation. We analogously estimate that both East Asian and Southeast Asian applicants had 17% lower odds of attendance, relative to similarly qualified white students. These gaps attenuate substantially after adjusting for the geographic distribution of applicants, and whether applicants are the children of alumni, suggesting these factors impact the likelihood of attendance. We note, however, that we do not have access to all materials submitted by and about applicants, such as essays, letters of recommendation, and alumni interviews, meaning we cannot fully account for applicants’ qualifications.

We conclude by exploring how the relative share of Asian American and white students at Ivy-11 colleges might change under various hypothetical admission policies. For this exercise, we assume that applicants who are admitted to one of the Ivy-11 schools we consider ultimately attend. Under a policy that admits students solely on the basis of standardized test scores and participation in extracurricular activities—and holding fixed the combined number of enrolled Asian American and white students—we estimate that enrollment of South Asian students and East Asian students would increase substantially, while the number of Southeast Asian students would remain approximately the same. These results complement existing studies that also show an increase in Asian American enrollment resulting from the hypothetical elimination of legacy preferences at selective schools^5,19^.

Concerns about the disparate impacts of college admissions policies on Asian American students are often entangled with discussions about affirmative action^2,9,20–26^. At their core, however, these two issues—affirmative action and differences in the admission rates of similarly qualified white and Asian American students—are conceptually distinct. In particular, during the time period we consider, institutions could have admitted Asian American applicants at rates comparable to similarly qualified white students while still giving preference to applicants from groups underrepresented in higher education. However, as of 2023, explicit racial preferences in college admissions are no longer legally permissible^27^.

By analyzing hundreds of thousands of recent applications to Ivy-11 colleges, our study expands on both the scale and scope of past investigations. Ours is also one of the first such studies to document disparities across Asian subgroups, revealing important differences within a population often treated as monolithic. We hope that these findings help inform the ongoing national conversation on the design of equitable college admissions policies and concomitant enrollment outcomes.

## Data description

Our analysis is based on applications submitted through a national postsecondary application platform. The data we use contain detailed, anonymized information on each student, including race and gender; standardized test scores (ACT and/or SAT); high school grade-point average (GPA); Advanced Placement (AP) exam scores; structured descriptions of their extracurricular activities (e.g., leadership positions and the number of hours they spent participating in various clubs or sports); the location and other characteristics of the high school they attended; whether their parents attended college, and, if so, the colleges they attended; whether they received an application fee waiver (which may be viewed as a proxy for financial need); the set of colleges to which they applied via the platform; and whether they applied early action or early decision to any of the institutions we consider (Table S4). If a student took the SAT, we convert their SAT score to an equivalent ACT score to facilitate comparisons between applicants and aid interpretation. If students took both the ACT and SAT, or took either test more than once, we choose the highest ACT-equivalent score achieved on a single test. Although we have quite detailed individual-level data, we do not have access to the full set of application materials, including student essays, letters of recommendation, or intended major. We also do not have access to internal college evaluations, such as interviewer ratings.

We infer attendance outcomes by observing the school to which a high school counselor sent a student’s official high school transcript, information that is collected by the platform. (NB: official transcripts typically are required by colleges to formalize enrollment.) We assessed the quality of our attendance inference by matching 5000 randomly selected applicants to the schools they actually attended, as reported by the National Student Clearinghouse. We find that the estimated precision of our attendance inference strategy is 97% with an estimated recall of 91%. We further find that accuracy is comparable across race groups (see “Methods”).

Our study pool is comprised of 685,709 applications submitted by 292,795 students to the 11 Ivy-11 colleges we consider in the 2015–2016 through the 2019–2020 application cycles. We include Asian and white applicants who attended a U.S. high school, excluding students from high schools for which we cannot reliably infer college enrollment (see “Methods” and Table S2). We cannot identify athletic recruits with certainty, but we exclude from our sample students who appear to be athletic recruits based on the timing of their inferred enrollment and their reported extracurricular activities (see “Methods”). Within our study pool, 36% of applicants self-identify as Asian, with 51%, 15%, and 34% of these students self-identifying as East Asian, Southeast Asian, and South Asian, respectively. Finally, we supplement our data from the platform with public high school data from the Common Core of Data (CCD), private high school data from the Private School Universe Survey (PSS), and rurality data at the ZIP code level from the Economic Research Service of the U.S. Department of Agriculture.

## Results

**Figure 1:**
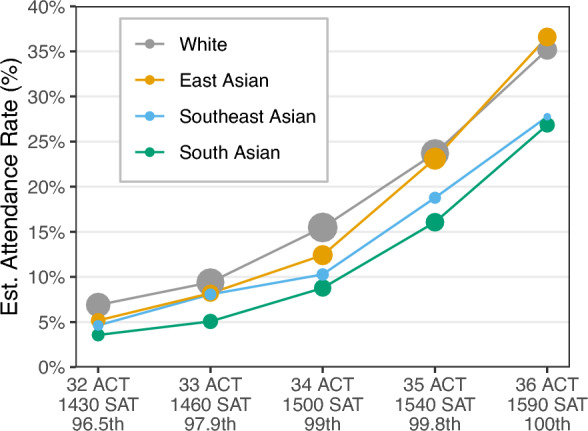
Estimated rate of attendance at any of the Ivy-11 colleges we consider as a function of standardized test score, for Asian American applicants and white applicants in the study pool. Asian American applicants typically attended at lower rates than white applicants with identical test scores, with the largest gap for South Asian students. Among the students in our study pool who attended an Ivy-11 and report ACT or SAT scores, 93% have ACT (or ACT-equivalent) scores at or above 32. Percentiles are derived from all students who took the ACT in 2018^28^. Point sizes are proportional to the number of applicants in each group.

Among applicants to the Ivy-11 colleges, we estimate that 16% of East Asian, 8% of Southeast Asian, and 10% of South Asian students ultimately attended one of these institutions, compared to 12% of white applicants. While these aggregate attendance rates differ by race and ethnicity, they do not account for differences in qualifications across groups. For example, Asian American applicants had, on average, higher standardized test scores than white applicants (Table S3). As a first step to account for these differences, in Fig. 1 we show estimated attendance rates by standardized test score for Asian American applicants and white applicants. With the exception of East Asian applicants at the highest test scores, we find that Asian American applicants to Ivy-11 schools ultimately attended one of these schools at consistently lower rates than white applicants with comparable test scores, with the largest gap for South Asian applicants. For instance, among applicants with an ACT (or ACT-equivalent) score of 34—placing them in the 99th percentile of test takers—we estimate that 16% of white applicants attended compared to 9% of South Asian applicants, a relative gap of 43%.

Standardized test scores are but one factor among many that impact admissions decisions and subsequent enrollment outcomes. Additional criteria that we are able to observe include high school grade-point average (GPA), participation in extracurricular activities, legacy status, and the state in which each applicant’s high school is located. To understand the extent to which these other considerations may explain the observed disparities in attendance rates, we fit a series of nested logistic regression models of the following form:

$$\begin{aligned}&\Pr (Y_i=1) = \text {logit}^{-1}( \beta _0\! +\! \beta _S \mathbbm {1}_S\! +\! \beta _E \mathbbm {1}_E\! +\! \beta _{SE} \mathbbm {1}_{SE}\! +\! X_i \beta _X ), \end{aligned}$$

where $Y_i$ is a binary variable indicating whether applicant *i* attended any Ivy-11 college; $\mathbbm {1}_S$, $\mathbbm {1}_E$, and $\mathbbm {1}_{SE}$ indicate whether the applicant identified as South Asian, East Asian, or Southeast Asian, respectively; and $X_i$ is a vector of additional covariates (e.g., test scores and GPA) that we vary across models, with $\beta _X$ the corresponding vector of coefficients. Our key coefficients of interest are $\beta _S$, $\beta _E$, and $\beta _{SE}$, which yield estimates of the gap in attendance rates between white applicants and Asian American applicants in the three Asian subgroups that we consider. We find similar results if we fit separate models comparing white applicants to applicants in each Asian subgroup individually (Tables S13–S15).

Table 1 shows, for nine models that include different subsets of control variables, the fitted coefficients for each of the three Asian subgroups (see also Tables S5–S12). Coefficients are exponentiated for ease of interpretation as odds ratios. The first model includes only fixed effects for the application season and the subset of colleges (or application “basket”) to which the student applied—among the full set of colleges we consider—facilitating comparisons among groups of students who applied in the same year and to the same subset of colleges. The corresponding coefficients are thus akin to raw attendance odds ratios across groups, without adjusting for differences in applicant credentials.

The second and third models in Table 1 additionally adjust for measures of academic preparation, including SAT/ACT alone (Model 2) and, additionally, GPA, AP test scores, and SAT II subject test scores (Model 3). These academic-preparation models corroborate the visual pattern in Figure 1: we estimate that Asian American students—especially South Asian students—had substantially lower odds of attendance than white students with similar test scores and related academic credentials. These disparities largely persist when we progressively adjust for extracurricular activities (Model 4); gender and family characteristics, like whether the student received an application fee waiver (Model 5); and whether the student applied early (Model 6).

**Table 1:** Estimated conditional odds of attendance at an Ivy-11 college for Asian American applicants in the study pool relative to white applicants.

	Outcome: inferred attendance at an Ivy-11 college								
	Basket + year	SAT/ACT	GPA + AP + SAT2	Activities	Sex + family	Early App	Legacy	Location + HS	All
	(1)	(2)	(3)	(4)	(5)	(6)	(7)	(8)	(9)
South Asian	0.66^∗∗∗^ (0.01)	0.56^∗∗∗^ (0.01)	0.59^∗∗∗^	0.51^∗∗∗^ (0.01)	0.51^∗∗∗^ (0.01)	0.52^∗∗∗^ (0.01)	0.61^∗∗∗^ (0.01)	0.60^∗∗∗^ (0.01)	0.70^∗∗∗^ (0.02)
			(0.01)						
Southeast Asian	0.64^∗∗∗^ (0.02)	0.73^∗∗∗^ (0.02)	0.78^∗∗∗^ (0.02)	0.83^∗∗∗^ (0.03)	0.81^∗∗∗^ (0.03)	0.84^∗∗∗^ (0.03)	0.88^∗∗∗^ (0.03)	0.94 (0.03)	1.02 (0.04)
East Asian	1.11^∗∗∗^ (0.02)	0.86^∗∗∗^ (0.01)	0.85^∗∗∗^ (0.01)	0.83^∗∗∗^ (0.02)	0.79^∗∗∗^ (0.01)	0.73^∗∗∗^ (0.01)	0.90^∗∗∗^ (0.02)	0.88^∗∗∗^ (0.02)	0.90^∗∗∗^ (0.02)
Aggregated Asian (separate model)	0.88^∗∗∗^ (0.01)	0.74^∗∗∗^ (0.01)	0.75^∗∗∗^ (0.01)	0.72^∗∗∗^ (0.01)	0.69^∗∗∗^ (0.01)	0.67^∗∗∗^ (0.01)	0.79^∗∗∗^ (0.01)	0.79^∗∗∗^ (0.01)	0.85^∗∗∗^ (0.02)
Basket + year	X	X	X	X	X	X	X	X	X
SAT/ACT		X	X	X	X	X	X	X	X
GPA + AP + SAT2			X	X	X	X	X	X	X
Activities				X	X	X	X	X	X
Sex + family					X	X	X	X	X
Early app						X			X
Legacy							X		X
Location + HS								X	X
Observations	292,795	292,795	292,795	292,795	292,795	292,795	292,795	292,795	292,795
In-sample AUC	0.65	0.75	0.79	0.81	0.82	0.85	0.83	0.84	0.88
Pseudo-R-squared	0.06	0.12	0.17	0.21	0.21	0.27	0.23	0.25	0.32
White base rate	12%	12%	12%	12%	12%	12%	12%	12%	12%

Coefficients are estimated via logistic regression, and are exponentiated for ease of interpretation as odds ratios. Pseudo-$\text {R}^2$ values between 0.2 and 0.4 indicate a well-fitted model^29^. After adjusting for test scores and extracurricular activities (Model 4), South Asian students had 48% lower estimated odds of attendance relative to white students, with East Asian and Southeast Asian applicants exhibiting smaller but statistically significant gaps (17% lower odds of attendance). These disparities appear to be explained in part by legacy preferences (Model 7) and geography (Model 8). The “Aggregated Asian” coefficients are computed from separate models that do not separate students into Asian subgroups.

^∗^p < 0.05; ^∗∗^p < 0.01; ^∗∗∗^p < 0.001

**Figure 2:**
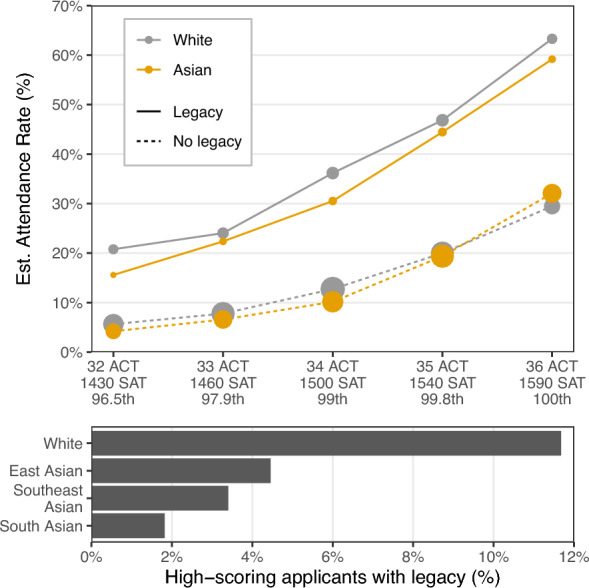
Estimated rate of attendance at any of the Ivy-11 colleges for white applicants and Asian American applicants with high ACT or SAT scores. Across test scores, we estimate that applicants with a parent who attended an Ivy-11 as an undergraduate are more than twice as likely to attend than non-legacy applicants with the same test scores. The bottom panel shows the proportion of applicants with high test scores who have legacy status, disaggregated by race. High-scoring white applicants are three to six times more likely to have legacy status than high-scoring Asian American applicants, suggesting white applicants disproportionately benefit from legacy status.

Next, with Model 7, we account for whether a student is the child of an alum. After adjusting for legacy status—in addition to all of the above mentioned factors—we see large reductions in the estimated disparities in attendance rates for all three Asian subgroups we consider. Figure 2 helps explain this result. The top panel of the figure shows estimated attendance rates for Asian American applicants and white applicants conditional on test scores and legacy status, which we define in this figure to mean an applicant had at least one parent who attended an Ivy-11 as an undergraduate, and the student applied to the Ivy-11 institution(s) that their parent(s) attended. For a given test score, we estimate that applicants—both white and Asian American—with legacy status at an Ivy-11 were more than twice as likely to attend an Ivy-11 than applicants without legacy status. In the bottom panel of Fig. 2, we present prevalence of legacy status among applicants with an ACT-equivalent test score of 32 or above, mirroring the focus of the upper panel. Here, we observe that white applicants were approximately three times more likely to have legacy status than East Asian and Southeast Asian applicants, and almost six times more likely than South Asian students. Thus, even though estimated attendance rates conditional on test score and legacy status are similar across race and ethnicity, white students appear to benefit from being substantially more likely to have legacy status.

The higher estimated attendance rates that we observe for legacy applicants may stem from either higher admission rates or higher yield rates. Though we can only speculate, it seems likely that both factors play a role. Further, to the extent that our results reflect disparities in admissions decisions, these findings may in theory be driven in part from the potentially greater social capital of legacy students, rather than explicit preferences for legacy applicants. We note, however, that Model 5 adjusts for whether an applicant had a parent who attended a top-50 institution (based on the 2019 U.S. News rankings) not included in the subset of colleges on which we focus, or attended an Ivy-11 college to which the student did not apply—proxies for having high social capital distinct from legacy status specifically. The change in disparities that we observe moving from Model 5 to Model 7 thus appears attributable to legacy status specifically, rather than the more generalized impacts of high social capital.

**Figure 3:**
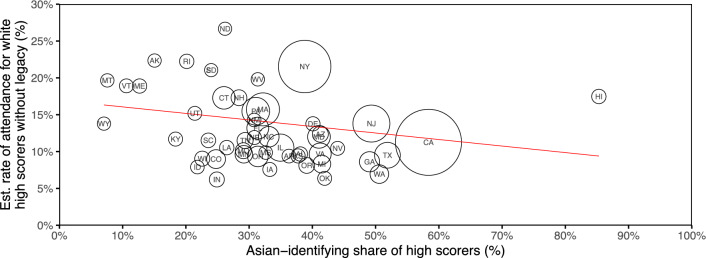
For each U.S. state, estimated rate of attendance at any Ivy-11 college for non-legacy white applicants with an ACT-equivalent score at or above 32, with the proportion of high-scoring white and Asian applicants who identify as Asian on the horizontal axis. We report attendance rates of non-legacy white applicants to better isolate the impact of geography on attendance from the potential impacts of legacy status and race itself. Larger point sizes indicate a higher number of high-scoring white and Asian applicants from the state. The red least-squares regression line is weighted by the same count of applicants. States with a greater share of Asian American applicants have, on average, lower estimated attendance rates for non-legacy white applicants with high scores.

Finally, we examine the relationship between estimated attendance rates and geography. For each state, Fig. 3 displays the estimated attendance rate of high-achieving applicants—with ACT-equivalent scores of 32 or above—to the fraction of applicants from that state who were Asian American. When computing attendance rates, we limit to non-legacy white applicants to adjust for the the potential effects of legacy and race on enrollment and therefore better isolate the impact of geography. Point sizes are proportional to the total number of high-scoring white and Asian American applicants in each state. The negatively sloped regression line shows that states with a larger fraction of Asian American applicants tended to have lower estimated attendance rates. Further, states with a higher proportion of Asian American applicants tended to have higher average test scores, suggesting the geographic trend is not driven by a gap in academic achievement (Fig. S2). This geographic pattern also persists when we exclude applicants from California, and when we disaggregate the data to the level of high school instead of state (Figs. S1 and S3). Table S27 displays the data used to construct Fig. 3.

Model 8 in Table 1—which adjusts for location as well as academic and extracurricular performance but not legacy status—shows that these apparent geographic preferences account for much of the attendance gap between white and Asian American applicants. Model 9, the last one we consider, adjusts for all application information available to us, including both legacy status and geography. After adjusting for this rich set of covariates, we see that the estimated attendance gap between Southeast Asian and white applicants largely disappears, though we still find that white applicants have higher estimated odds of attendance than otherwise similar East Asian and South Asian applicants. It is unclear what may account for these remaining disparities, though it bears repeating that admissions officers have access to more complete application materials than do we—including letters of recommendation, essays, and interview assessments—and student enrollment choices may also be impacted by factors unobserved in our data.

We conclude our analysis by exploring how the relative share of Asian American students at the institutions we consider might change under various hypothetical admissions policies. To simplify this exercise, we assume that students admitted to an Ivy-11 school ultimately attend an Ivy-11 school. In line with our analysis above, we restrict our attention to white students and Asian American students. Specifically, we hold fixed the combined number of students in these groups (approximately mirroring historical enrollment, as shown in Fig. S4), and so any increases in Asian American enrollment necessarily imply decreases in enrollment of white students. Any exercise of this sort is inherently speculative—in part because changes in admissions policies could alter application behavior—but we still believe it is informative to gauge the approximate magnitude of effects.

**Figure 4:**
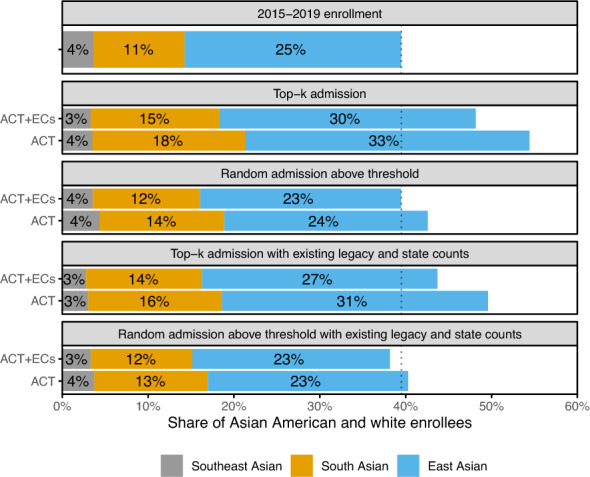
Estimated enrollment of Asian American students at the Ivy-11 under eight hypothetical admissions policies, with the top panel showing the actually observed demographic composition in our historical data. In all cases, we consider only the subset of Asian American students and white students, and so increases in Asian American enrollment correspond to decreases in the enrollment of white students. In most instances, the hypothetical policies we consider lead to an increase in enrollment of Asian American students, including those that preserve the number of legacy students and the number of enrollees from each state in the historical data.

As a baseline, the top row of Fig. 4 shows the estimated share of attendees in our data from the three Asian subgroups of interest. The rest of the figure shows the estimated share of attendees from these subgroups under eight hypothetical admissions policies that are divided into four categories. In the first category—which we call “top-*k*” policies—we imagine admitting students with the highest ACT-equivalent scores, with ties broken randomly. In the second category, “random above threshold,” we consider policies that randomly admit students above an ACT-equivalent score *t* such that admitted students have a mean score equal to that of actual enrollees^30^. For both of these categories we consider two variants: the “ACT” variant selects from the entire applicant pool of the schools we consider, while the “ACT+ECs” variant selects only from applicants with at least as many hours of reported extracurricular (EC) activities over four years of high school as the median of the hours reported by all enrollees. Under all four policies, we estimate the same or larger shares of Asian American students compared to what we observe in the data. Asian American students report, on average, fewer extracurricular hours than white applicants, so the ACT+ECs policy variant results in fewer Asian American attendees than the ACT variant.

The final two categories we consider investigate outcomes under hypothetical policies that maintain both the current number of attendees from each state and the total number of attendees with legacy. Specifically, we first divide our historical data into 102 (2 x 51) cells consisting of legacy and non-legacy applicants from each U.S. state and Washington, D.C.; we then in turn apply each of the four policies described above to each of the 102 cells, ensuring for each cell that the number of students admitted under the hypothetical policies matches the historical enrollment numbers. With these added legacy and geographic constraints, the share of Asian American attendees is smaller than under the unconstrained analogs, as expected given our results above. But, even with these constraints, the number of Asian American attendees across policies is still similar to or larger than the status quo.

## Discussion

Based on a large-scale analysis of applications to 11 highly selective colleges, we find that Asian American students were less likely to ultimately attend one of these schools than white students with comparable academic credentials and extracurricular activities, a disparity that is particularly pronounced for South Asian applicants. It further appears that much—though not all—of this gap is attributable to geography and legacy status. In particular, applicants from parts of the country with lower shares of Asians, and applicants with legacy status, who are disproportionately white, attend these selective schools at higher rates than similarly qualified Asian Americans applicants.

While our main findings focus on Ivy-11 colleges, we replicate our analysis using an expanded set of selective colleges and find qualitatively similar, though somewhat attenuated, results (see Table S17). Further, in our primary analysis, we excluded applicants who we inferred were recruited athletes, under the assumption that filling sports teams is a hard constraint for many universities, and that doing so involves qualitatively distinct admissions criteria. We note, though, that athletic recruits are disproportionately likely to be white rather than Asian American: in our study pool, white applicants outnumber Asian American applicants by a factor of about two to one, but among inferred recruits, white applicants outnumber Asian American applicants by a factor of four to one. As a result, if we do not proactively exclude recruited athletes from our analysis, we find an even larger gap in the estimated enrollment rates between Asian American students and white students with comparable academic credentials (Tables S13–S15). We also note that our analysis concerns disparities with respect to Asian American applicants, so we exclude international applicants. Asian applicants make up a disproportionately large share of international applicants relative to white applicants (Fig. S7). The disparities we observe could differ if international applicants were also considered. However, we do not observe reliable signals of attendance for international applicants, so we cannot investigate this possibility.

The attendance outcomes we study are a product of both the admissions decisions of colleges and the enrollment choices of students. Our data do not allow us to cleanly disentangle these two distinct mechanisms, though there is reason to believe that both contribute to the disparities we observe. For example, prior research has found that admits with legacy status are more likely to enroll than non-legacy admits^31^, highlighting the role of student decisions in attendance outcomes. On the other hand, there are several reasons to believe that the disparities we see are driven at least in part by the admissions choices of colleges. First, Ivy-11 colleges have among the highest matriculation rates, highlighting the value that admitted students place on attending the schools we study. Indeed, among the ten institutions with the highest matriculation rates that enroll at least 1000 first-year students, nine are included in our analysis^17^. In Table S16, we replicate the main analysis using only applications to Ivy-11 schools with yield rates above 80%, finding qualitatively similar gaps in attendance rates for South and East Asian applicants. Second, all schools in the Ivy-11 meet 100% of the demonstrated financial need of admits, eliminating a common reason—lack of affordability—for students not to attend a college to which they were admitted. Finally, we note that the disparities in attendance outcomes that we find are generally similar to reported disparities in admissions decisions at Harvard, recently made public as part of the Harvard v. SFFA court case. It thus seems unlikely that differential enrollment choices among admitted students are solely responsible for the disparities in attendance that we observe.

One important limitation of our work is that we do not have access to each student’s complete application materials. In particular, we do not observe a student’s essays, teacher and counselor recommendation letters, transcripts, and interview ratings. It is thus possible that students who appear to have similar academic and extracurricular credentials are in fact differentially qualified in ways revealed in these other materials. We note, however, that prior work has found that Asian American and white college applicants with similar academic credentials receive letters of recommendation from teachers that are broadly similar in content and tone^23^. Further, turning again to results made public through litigation with Harvard, the disparities we observe persist after adjusting for additional markers of academic and extracurricular excellence, including admission officer ratings of each applicant’s academics and teacher and counselor recommendations—at least in the case of Harvard’s admissions practices^32^.

Discussions of college admissions practices impacting Asian Americans often revolve around affirmative action. But, as we noted at the start, these issues are conceptually distinct. In theory, one can both implement affirmative action policies that maintain the share of students on campus from groups that are underrepresented in higher education while simultaneously admitting and enrolling Asian American students at the same rate as white students with similar academic and extracurricular credentials. In such a case, we would expect the number of enrolled white students to decrease, not the number of racial minorities. During the time period we examined, affirmative action was widely used for shaping the diversity of college campuses, meaning the scenario described above was an option available to college administrators. Thus, at the very least, our results shed light on past choices and their consequences for Asian American college applicants.

With the end of affirmative action, institutions will need to reconsider how applicants are evaluated in order to ensure equitable admissions processes and to maintain diverse campuses. Our work highlights one potentially important direction for attaining those goals: reevaluating admissions policies that favor the children of alumni. Existing policies that afford preference to legacy applicants appear to not only disadvantage Asian Americans but also other racial minorities (Fig. S5). Further, legacy status at selective schools across the country tends to be concentrated among white applicants (Fig. S6), suggesting that legacy preferences have equity implications beyond the Ivy-11 schools we primarily focus on. Looking ahead, we hope our findings facilitate ongoing discussions about the design and implementation of equitable admissions policies.

## Methods

### Data filtering

We begin our core analysis with the 551,292 South Asian, East Asian, Southeast Asian, and white students who submitted at least one application to an Ivy-11 via the national postsecondary application platform in the 2015–2016 application cycle through the 2019–2020 cycle. We then filter to the 449,564 applicants who attended a high school in a U.S. state or the District of Columbia, and who did not report citizenship outside of the United States. We next limit to the 297,417 applicants who attended high schools with official transcript sends that, to the best of our knowledge, accurately reflect an intention to attend. Finally, for our main analysis, we restrict to the 292,795 applicants who, to the best of our knowledge, are not athletic recruits.

### Identifying high schools with reliable transcript sending behavior

Typically, when an applicant intends to enroll in a college to which they were admitted, their high school must submit an official transcript to the college. Many high schools use the same portal to submit official transcripts, and the platform observes when an applicant’s transcript is sent via this portal. While the platform does not observe acceptances or enrollments, high school transcript sends serve as a highly accurate enrollment proxy for the subset of applicants who meet the following conditions:

- First, the applicant’s high school must use the transcript sending platform. In other words, we only include applicants whose high school sent at least one transcript via the platform in the same year the applicant applied.
- Second, transcript sends must be targeted to specific colleges. If a high school counselor does not track the intended enrollment of a particular applicant, they may indiscriminately send final transcripts to every college to which the applicant submitted an application.

We define a high school’s transcript sending behavior as “reliable” in a given year if the high school submits the same number of transcripts as applications for fewer than 5% of applicants who submit at least two applications. In computing this proportion, we do not consider applicants who submit one application and one transcript, as we cannot reliably guess whether the student intended to enroll or the transcript was sent indiscriminately by their counselor. In Tables S13–S15, we replicate the main results with thresholds other than 5%, finding qualitatively similar results.

Among the applicants from high schools who exhibit reliable transcript sending behavior, we further exclude the applicants who submitted the same number of transcripts as applications and submitted at least two applications, since these students’ counselors likely sent the transcripts indiscriminately.

### Verifying transcript send enrollment signal with NSC data

Using a stratified random sample of 5000 enrollments obtained from the National Student Clearinghouse (NSC), we find that our enrollment heuristic based on transcripts has nearly perfect precision (97%) and high, but not perfect, recall (91%). The 5000 sampled applicants were selected from the study pool, which includes only those applicants whose high school met the threshold for reliable transcript sending behavior in the given application year.

Among the first stratum of 2500 applicants with a transcript sent to at least one of the Ivy-11 schools, we were able to match 2336 (93%) to NSC enrollments. We find that 2271 actually attended an Ivy-11 within a year of admission. Thus, the estimated precision of the enrollment proxy is 97%. Precision is nearly identical across race groups: white students have a precision of 97%, while Asian American students have a precision of 97.1%. Precision is also similar across application years, high school states, and application fee waiver status.

For the second stratum of 2500 applicants who applied to an Ivy-11 college but did not send a transcript to an Ivy-11 college, we were able to match 2294 (92%) to NSC enrollments. 43 (2%) of the 2294 matched applicants ended up attending an Ivy-11 college within a year of applying. We attribute this discrepancy to a number of potential factors: students may be admitted off the waitlist at an Ivy-11 college; individual counselors may not use the transcript sending portal even if the rest of the high school uses the portal; or students may transfer to an Ivy-11 college after initially being rejected. To determine the source of the discrepancy, we disaggregate by whether the matched applicant sent a transcript to any school on the platform.

1247 of the 2294 matched applicants sent a transcript to a school on the platform outside of the Ivy-11. Of these 1247 applicants, 13 actually ended up attending an Ivy-11 within a year of applying, but 10 of these 13 first attended the school to which they sent a transcript. We assume that these 10 students transferred to one of the Ivy-11 colleges after initially being rejected, so we exclude them from the error calculation, as our proxy for attendance is assumed to be correct for the year their observed applications were submitted. The remaining three applicants may have been admitted off the waitlist at one of the Ivy-11 schools after initially committing to a different school. In the study pool, 139,888 applicants sent a transcript to a school that was not an Ivy-11. Thus, from these applicants, we estimate 139,888*(3/1247) = 337 unobserved enrollments at the Ivy-11.

The remaining 1057 of the 2294 matched applicants did not send a transcript to any school. 30 (3%) of the 1057 applicants ended up at an Ivy-11. We attribute these 30 enrollments to the idiosyncratic counselor behavior described above. In the study pool, 117,138 applicants did not send a transcript to any school on the platform. Thus, we estimate 117,138*(30/1057) = 3325 unobserved enrollments from these applicants.

In sum, in the study pool, we observe 35,769 enrollments at Ivy-11 colleges. We estimate 337 + 3325 = 3662 unobserved enrollments. Thus, our estimated recall is 35,769/(35,769 + 3662) = 91%. Given that our estimated recall is based on discrepant enrollments of only 33 matched applicants, we cannot meaningfully evaluate recall across groups.

### Identifying potential athletic recruits

In order to field competitive athletic teams, universities often recruit students with exceptional athletic ability. University admission offices may have a hard constraint of filling athletic teams with a sufficient number of talented student athletes. Admissions decisions for student athletes are primarily the choice of athletic coaches, who are incentivized to offer admission to recruits with the greatest athletic ability who meet or exceed the minimum academic qualifications for admission^33,34^. Further, student athletes are typically admitted early^35^. We thus attempt to exclude students who, to our knowledge, may be athletic recruits, as the admission process for student athletes differs considerably from that of typical applicants. As a robustness check, we repeat the main analysis without excluding potential recruits, finding qualitatively similar results (Tables S13–S15).

While we do not observe true athletic recruitment status, we have access to detailed information about each applicant’s extracurricular participation. We know not only the extracurricular activities of each applicant, but also the number of years participated in each activity during high school, the order in which those activities are reported in their application, and whether the applicant intends to continue participating in the activity in college. We assume that student athletes will list their athletic participation as the first activity in their application. We further assume that student athletes will have participated in their first-listed sport during all four years of high school, and that they intend to continue participating in their first-listed sport in college. Finally, we assume that student athletes will only apply to one college or university in an early round, and that they always send a transcript to that one institution (i.e., attend). Thus, all inferred recruits have a record of attendance at the Ivy-11 to which they applied early, and they did not apply anywhere else.

Among the 40,391 white and Asian American applicants who submitted a transcript to an Ivy-11 college and who also attended a high school in the U.S. with reliable transcript-sending behavior, 4622 applicants (11%) were potential recruits according to our definition. While we cannot formally verify that these students were actually recruited by an Ivy-11 college, 11% is in line with public estimates of the fraction of selective university enrollees who are student athletes^35^. Additionally, among the 17,373 applicants who enrolled in an Ivy-11 to which they applied early and who applied to only one Ivy-11, 27% meet the remaining requirements of our definition of potential recruit (i.e., four years of participation, intention to continue in college, and listing a sport as the first extracurricular), compared to 14% of the remaining 23,018 Ivy-11 enrollees. This finding is consistent with a plausible (though unverifiable) scenario in which recruits are more likely than non-recruits to report an athletics-related activity first in their list of extracurriculars.

### Supplementary Information

Supplementary Information.

## Data Availability

We provide aggregated data that can be used to replicate our main results (cf. Tables S20–S26 in the Appendix). To protect the anonymity of applicants, we are not releasing the raw, applicant-level data. Code to reproduce our analysis and aggregated data are available for download at: https://github.com/joshuagrossman/asian-admission-di.
