# Supplementary material for: The disparate impacts of college admissions policies on Asian American applicants
Source: Sci Rep. 2024 Feb 23;14:4449. doi: 10.1038/s41598-024-55119-0 (PMC10891048; doi:10.1038/s41598-024-55119-0)
Supplement: Supplementary file 1 — Supplementary Information. [file 41598_2024_55119_MOESM1_ESM.pdf]

Supplementary Information for “The Disparate Impacts of College Admissions Policies on Asian American Applicants”

Joshua Grossman[1,*], Sabina Tomkins[2], Lindsay Page[3], and Sharad Goel[4]

[1]Stanford University, Department of Management Science and Engineering, Stanford, 94305, USA
[2]University of Michigan, School of Information, Ann Arbor, 48109, USA
[3]Brown University, Education Department, Providence, 02912, USA
[4]Harvard University, Kennedy School of Government, Cambridge, 02138, USA
[*]jdgg@stanford.edu

Table of contents

Estimating Attendance Rates summarizes a complementary analysis contingent on the weaker assumption that attendance is independent of race, conditional on acceptance and other observed student characteristics.

Table S1 shows the proportion of publicly reported applications reported by the institutions in the main analysis that were submitted via the national postsecondary application platform.

Table S2 shows summary statistics for applicants from high schools with reliable and unreliable records of sent transcripts.

Table S3 shows summary statistics for white, East Asian, South Asian, and Southeast Asian applicants in the study pool.

Table S4 lists relevant covariates observed and unobserved by the authors and by the application platform.

Tables S5 through S12 list and describe the covariates used in the main model specifications.

Tables S13 through S15 describe robustness checks of the main model specification.

Table S17 replicates the main results using an expanded set of selective schools.

Table S18 replicates the main results using an interaction between ethnicity and reported sex.

Tables S20 through S26 show the number of applicants in various coarsened groups defined by race and ethnicity, test score, legacy status, region of the country, and enrollment signal.

Table S27 displays the data used to construct Figures 3, S1, and S2.

Figures S1 through S3 show robustness checks of the state-level analysis in the main text.

Figures S4 and S5 support the hypothetical admissions policy results in the main text.

Figure S6 shows differences in legacy prevalence among students of different race and ethnic groups across a large group of selective schools.

Figure S7 displays the ethnicity of high-scoring white- and Asian-identifying applicants, disaggregated by international applicant status.

Figure S8 shows how often parents attended international or U.S.-based colleges, disaggregated by ethnicity.

Estimating Attendance Rates

In the Discussion, we speculate that students who were admitted to an Ivy-11 would have a high probability of attendance. In this way, one might infer admissions decisions from reported attendance.—which we can in turn accurately impute by looking at the institution to which a student sent their final high school transcript. Here we describe an alternative estimation strategy that holds under the weaker assumption that enrollment choices are independent of race conditional on acceptance and other observable student characteristics.

Denote by A the event that a particular applicant is admitted to an Ivy-11, where $A = 1$ if the applicant is admitted and $A = 0$ if the applicant is not admitted. Denote by E the analogous attendance event. Finally, denote by R the race of the applicant, and by W a set of non-race covariates. Now, suppose we are interested in comparing the admission probability of an applicant with race R and another applicant of race R' with identical non-race covariates W. We can express this comparison as a risk ratio:

$$\frac{\Pr(A=1 \mid W,R')}{\Pr(A=1 \mid W,R)}.$$

Without observing admission outcomes, the above ratio cannot be estimated directly. But, suppose we assume that $E \perp\!\!\!\perp R \mid A = 1, W$. In other words, conditional on acceptance and all observed non-race covariates, the decision to attend an Ivy-11 schools is independent of race. Then,

$$\begin{aligned}\Pr(E=1 \mid W,R) &= \Pr(A=1 \mid W,R) \cdot \Pr(E=1 \mid A=1,W,R)\\ &= \Pr(A=1 \mid W,R) \cdot \Pr(E=1 \mid A=1,W),\end{aligned}$$

and so

$$\Pr(A=1 \mid W,R) = \frac{\Pr(E=1 \mid W,R)}{\Pr(E=1 \mid A=1,W)}.$$

Applying this result to the acceptance risk ratio:

$$\begin{aligned}\frac{\Pr(A=1 \mid W,R')}{\Pr(A=1 \mid W,R)} &= \frac{\Pr(E=1 \mid W,R')}{\Pr(E=1 \mid A=1,W)} \cdot \frac{\Pr(E=1 \mid A=1,W)}{\Pr(E=1 \mid W,R)}\\ &= \frac{\Pr(E=1 \mid W,R')}{\Pr(E=1 \mid W,R)}.\end{aligned}$$

Thus, by assuming that attendance is independent of race conditional on acceptance and non-race covariates, we can estimate the acceptance ratio using only data on attendance. Averaging over W, we have

$$\sum_W \frac{\Pr(A=1 \mid W,R')}{\Pr(A=1 \mid W,R)} \cdot \Pr(W) = \sum_W \frac{\Pr(E=1 \mid W,R')}{\Pr(E=1 \mid W,R)} \cdot \Pr(W). \quad (1)$$

Importantly, the right-hand side of Eq. (1) can be estimated directly from records of attendance, as done with the main models in our analysis (Table 1). In particular, take R to be white students and R' to be, in turn, the three Asian subgroups we consider. Then, after adjusting for test scores, GPA, and extracurricular activities (i.e., by using Model 4 in the main text), we estimate that the average acceptance ratio is 0.58 for South Asian applicants, 0.85 for East Asian applicants, and 0.89 for Southeast Asian applicants. These estimates align with the results we report in Table 1, corroborating our main analysis.

	Academic year	Proportion
1	2015-2016	99%
2	2016-2017	98%
3	2017-2018	96%
4	2018-2019	93%
5	2019-2020	92%

Table S1. Approximate proportion of all publicly reported applications to Ivy-11 colleges that were submitted via the application platform, by academic season. The share of applications submitted via the platform has decreased in recent years as alternative platforms have become more popular.

Variable	All	Included	Excluded
Tot. applicants	444,420	292,795	151,625
Prop. sent transcript	10%	12%	7%
Prop. white	64%	64%	66%
Prop. Asian American	36%	36%	34%
Prop. East Asian	17%	18%	15%
Prop. South Asian	12%	12%	12%
Prop. Southeast Asian	6%	6%	8%
Mean num. apps submitted anywhere	7.9	8.4	7.1
Mean num. apps submitted to subset	2.3	2.3	2.2
Prop. applied early	39%	42%	34%
Prop. w/ legacy	6%	7%	3%
Mean ACT score	32.1	32.4	31.4
Prop. unreported ACT	14%	14%	13%
Mean standardized GPA	0.8	0.8	0.8
Prop. unreported GPA	14%	16%	10%
Mean num. AP tests	4	4.1	3.8
Median activity hours	3196	3236	3107
Median sports hours	480	540	400
Prop. female	53%	53%	54%
Prop. first generation	14%	12%	19%
Prop. using fee waiver	15%	12%	20%
Prop. rural HS	6%	4%	9%
Prop. private HS	22%	27%	13%
Median grad. class size	333	319	366
Prop. from California	17%	16%	19%
Prop. from Texas	5%	4%	6%
Prop. from Florida	3%	3%	4%
Prop. from New York	13%	15%	9%

Table S2. Summary statistics for the 'Included' applicants who attend high schools with reliable transcript-sending behavior, the 'Excluded' applicants who do not, and the combined set of 'All' applicants. On average, the 'Included' applicants submit more applications, apply early with a greater likelihood, are more likely to have legacy status, have higher standardized test scores, have more extracurricular hours, are more likely to play sports, are less likely to use application fee waivers, are more likely to attend urban and private high schools, and have smaller graduating class sizes. We re-run the main regression by inversely weighting the probability that a given applicant attends a high school with reliable transcript behavior, finding qualitatively similar results (Tables S13-S15, 'Reweighted' model variant).

Variable	All	White	Asian	E Asian	S Asian	SE Asian
Tot. applicants	292,795	186,079	106,716	53,856	36,389	16,471
Prop. sent transcript	12%	12%	13%	16%	10%	8%
Prop. white	64%	100%	0%	0%	0%	0%
Prop. Asian American	36%	0%	100%	100%	100%	100%
Prop. East Asian	18%	0%	50%	100%	0%	0%
Prop. South Asian	12%	0%	34%	0%	100%	0%
Prop. Southeast Asian	6%	0%	15%	0%	0%	100%
Mean num. apps submitted anywhere	8.4	8.1	9	9	9.5	7.7
Mean num. apps submitted to subset	2.3	2.1	2.8	3	2.9	2.3
Prop. applied early	42%	41%	44%	50%	42%	33%
Prop. w/ legacy	7%	10%	3%	4%	2%	3%
Mean ACT score	32.4	32.2	32.9	33.3	32.8	31.4
Prop. unreported ACT	14%	17%	10%	9%	11%	11%
Mean standardized GPA	0.8	0.8	0.8	0.8	0.7	0.8
Prop. unreported GPA	16%	16%	15%	16%	15%	14%
Mean num. AP tests	4.1	3.6	5	5.3	5.1	4
Median activity hours	3236	3384	2975	3131.7	2862	2688
Median sports hours	540	728	240	318	162	240
Prop. female	53%	52%	53%	54%	51%	56%
Prop. first generation	12%	9%	16%	18%	10%	25%
Prop. using fee waiver	12%	8%	19%	19%	14%	30%
Prop. rural HS	4%	5%	1%	2%	1%	2%
Prop. private HS	27%	31%	20%	20%	17%	23%
Median grad. class size	319	280	400	400	403	372
Prop. from California	16%	11%	24%	26%	16%	31%
Prop. from Texas	4%	3%	5%	4%	7%	5%
Prop. from Florida	3%	3%	2%	1%	3%	3%
Prop. from New York	15%	14%	15%	17%	13%	12%

Table S3. Summary statistics for the race and ethnic groups included in the analysis. White applicants are more likely to have legacy status than Asian applicants, have a greater number of extracurricular hours, on average, and are more likely to attend smaller and private high schools. East and South Asian applicants have, on average, higher standardized test scores and take more AP tests than white and Southeast Asian applicants.

	Platform and our data	Platform, but not our data	Neither platform nor our data
1	Unique applicant identifier	Full name	Athletic recruitment eligibility
2	Gender	High school transcript(s)	True admission outcome(s)
3	Race, ethnicity, and region(s) of origin	Academic honors	True enrollment outcome(s)
4	Age	Letters of recommendation	Ratings of admission officers
5	Citizenship status	Essays and written responses	Alumni interview ratings
6	High school name and location	Intended career	Official test scores
7	High school graduation date	College-specific fields (e.g., major)	Family income and assets
8	Self-reported test scores		
9	Self-reported GPA, GPA weighting, and class rank		
10	Highest educational attainment of parents		
11	Institutions attended and degrees obtained by parents		
12	Extracurricular categories, years participated, hours participated per year, leadership positions, and free text description		
13	Application submission status at individual colleges		
14	Application timing (e.g., restrictive early action)		
15	Application fee waiver status at individual colleges		
16	Receipt(s) of official transcript submission to individual colleges sent via the platform		

Table S4. Variables observed by the national postsecondary application platform and the authors, only the platform, and neither the platform nor the authors.

	Covariate	Additional description
1	Intercept	
2	South Asian	Applicant identifies as South Asian
3	Southeast Asian	Applicant identifies as Southeast Asian
4	East Asian	Applicant identifies as East Asian
5	Year by college fixed effects	Term for each combination of selective college applied to and application year, e.g., 'College X 2016'

Table S5. Variables includes in Model 1, 'Basket+year'.

	Covariate	Additional description
1	Equivalent ACT Composite Score	If SAT score reported, converted to equivalent ACT score
2	Equivalent ACT Composite Score Squared	
3	Missing ACT Score	Student did not report an ACT or SAT score

Table S6. Variables included in Model 2, 'SAT/ACT'. Variables from Model 1 are also included.

	Covariate	Additional description
1	Standardized GPA	GPA standardized by high school and year
2	Missing Cumulative GPA	Student did not report a GPA
3	Standardized ACT	
4	Std. Num. AP	Standardized number of AP tests taken
5	Std. Num. Passed AP	Standardized number of AP tests with a reported score of 3 or higher
6	Std. Num. 5 AP	Standardized number of AP tests with a reported score of 5 (maximum)
7	Std. Num. SAT Subject	
8	Std. Num. SAT Subject 700	Standardized number of SAT subject tests with a score of at least 700
9	Std. Num. Science AP	
10	Std. Num. History AP	
11	Std. Num. Math AP	
12	Std. Num. English AP	
13	Std. Num. Language AP	
14	Std. Num. Social Science AP	
15	Std. Num. Arts AP	
16	Std. Num. Science SAT Subject	
17	Std. Num. History SAT Subject	
18	Std. Num. Math SAT Subject	
19	Std. Num. English SAT Subject	
20	Std. Num. Language SAT Subject	
21	Took Art Studio Art 2D Design AP	
22	Took Art Studio Art 3D Design AP	
23	Took Art Studio Art Drawing AP	
24	Took Biology AP	
25	Took Biology Ecological SAT Subject	
26	Took Biology Molecular SAT Subject	
27	Took Calculus AB AP	
28	Took Calculus BC AP	
29	Took Calculus BC AB Subscore Grade AP	Reported a Calculus AB subscore for AP Calculus BC
30	Took Chemistry AP	
31	Took Chemistry SAT Subject	
32	Took Computer Science A AP	
33	Took Economics Macroeconomics AP	
34	Took Economics Microeconomics AP	
35	Took English Language Composition AP	
36	Took English Literature Composition AP	
37	Took Environmental Science AP	
38	Took European History AP	
39	Took French Language AP	
40	Took French Reading SAT Subject	
41	Took French With Listening SAT Subject	
42	Took German Language AP	
43	Took German Reading SAT Subject	
44	Took German With Listening SAT Subject	

45	Took Government Politics Comparative AP	
46	Took Government Politics United States AP	
47	Took History Of Art AP	
48	Took Human Geography AP	
49	Took Italian Language Culture AP	
50	Took Italian Reading SAT Subject	
51	Took Latin AP	
52	Took Latin Reading SAT Subject	
53	Took Latin Literature AP	
54	Took Latin Vergil AP	
55	Took Literature SAT Subject	
56	Took Math Level 1 SAT Subject	
57	Took Math Level 2 SAT Subject	
58	Took Music Theory AP	
59	Took Music Theory Aural Subscore AP	
60	Took Music Theory Nonaural Subscore AP	
61	Took Physics SAT Subject	
62	Took Physics 1 AP	
63	Took Physics 2 AP	
64	Took Physics B AP	
65	Took Physics C Electricity Magnetism AP	
66	Took Physics C Mechanics AP	
67	Took Psychology AP	
68	Took Research AP	
69	Took Seminar AP	
70	Took Spanish Language AP	
71	Took Spanish Literature AP	
72	Took Spanish Reading SAT Subject	
73	Took Spanish With Listening SAT Subject	
74	Took Statistics AP	
75	Took US History SAT Subject	
76	Took United States History AP	
77	Took World History AP	
78	Took World History SAT Subject	
79	Took Writing SAT Subject	

Table S7. Variables included in Model 3, ‘GPA+AP+SAT2’. Variables from all prior model are also included. Standardization is by high school-year using all applicants observed by the platform. We standardize by subtracting the sample mean and dividing by the sample standard deviation. Standardized values for high school-years with only one observation are coded as 0. Standardized values are capped at 3 and floored at -3.

	Covariate	Additional description
1	Archery	8 covariates per sport: Log total number of hours participated in sport, binary indicator for leadership role in sport, binary indicator for four years of high school participation in sport, and binary indicator for leadership and four year participation in sport, with separate covariates for JV/Varsity participation and Club participation
2	Badminton	
3	Baseball	
4	Basketball	
5	Bowling	
6	Boxing	
7	Cheerleading	
8	Cricket	
9	Crosscountry	
10	Diving	
11	Equestrian	
12	Fencing	
13	Field Hockey	
14	Football	
15	Golf	
16	Gymnastics	
17	Handball	
18	Ice Hockey	
19	Indoor Track	
20	Judo	
21	Lacrosse	
22	Other Sport	
23	Outdoor Track	
24	Racquetball	
25	Rifle	
26	Rowing Crew	
27	Rugby	
28	Sailing	
29	Skiing	
30	Soccer	
31	Softball	
32	Squash	
33	Swim	
34	Sync swimming	
35	Table Tennis	
36	Tennis	
37	Track and field	
38	Triathlon	
39	Volleyball	
40	Water polo	
41	Weight lifting	

42	Wrestling	
43	Academic	4 covariates per activity type: Identical to sports, but without the JV/Varsity or Club designation
44	Art	
45	Career Oriented	
46	Volunteering	
47	Computer/Technology	
48	Cultural	
49	Dance	
50	Debate/Speech	
51	Environmental	
52	Family Responsibilities	
53	Foreign Exchange	
54	Foreign Language	
55	Journalism/Publication	
56	Junior ROTC	
57	LGBT	
58	Music Instrumental	
59	Music Vocal	
60	Other Activity	
61	Religious	
62	Research	
63	Robotics	
64	School Spirit	
65	Science/Math	
66	Student Govt/Politics	
67	Theater/Drama	
68	Work Paid	

Table S8. Variables included in Model 4, 'Activities'. Variables from all prior model are also included.

	Covariate	Additional description
1	Male	
2	Received Platform Fee Waiver	Received an income-eligibility fee waiver for any school applied to via the platform
3	Received Subset Platform Fee Waiver	Received an income-eligbility fee waiver at any Ivy-11 college
4	Received Subset Member Fee Waiver	Received a fee waiver directly from the considered school (not necessarily related to income)
5	Highest Parent Educ. is High School	
6	Highest Parent Educ. is Some College	
7	Highest Parent Educ. is 4 year College Degree	
8	Highest Parent Educ. is Graduate School	
9	Highest Parent Educ. is Unknown	
10	Top 50 Non Subset Legacy Undergrad 1	First listed parent attended a Top 50 university defined by U.S. News in 2019 outside of the Ivy-11 as an undergraduate
11	Top 50 Non Subset Legacy Undergrad 2	
12	Top 50 Non Subset Legacy Grad 1	
13	Top 50 Non Subset Legacy Grad 2	
14	No App Subset Legacy Undergrad	Either parent was an undergraduate at a considered schools that the applicant did not apply to
15	No App Subset Legacy Grad	

Table S9. Variables included in Model 5, ‘Sex+Family’. Variables from all prior model are also included.

	Covariate	Additional description
1	Early Application Subset	Applied to an Ivy-11 college under restrictive early action
2	Early Decision Subset	

Table S10. Variables included in Model 6, ‘Early App’. Variables from Models 1 through 5 are also included.

	Covariate	Additional description
1	Subset Double Legacy Undergrad	Both parents were undergraduates at the same Ivy-11 college to which the student applied
2	Subset Double Legacy Grad	
3	Subset Double Legacy Mixed	One parent was an undergraduate and the other parent was a graduate student at the same Ivy-11 college to which the student applied
4	Subset Single Legacy Undergrad	Exactly one parent was an undergraduate at a considered school to which the student applied
5	Subset Single Legacy Grad	
6	Subset Two Separate Legacy Undergrad	Each parent was an undergraduate at an Ivy-11 college to which the student applied and both attended a different Ivy-11 college
7	Subset Two Separate Legacy Grad	

Table S11. Variables included in Model 7, 'Legacy'. Variables from Models 1 through 5 are also included.

	Covariate	Additional description
1	Log Graduating Class Size	
2	Prop. Students Applying Platform	Proportion of students in the graduating class who submitted at least one application via the platform
3	Prop. Free Reduced Lunch	
4	Missing Prop. Free Reduced Lunch	Unknown proportion of students receiving free or reduced lunch in high school
5	Is Private	Attended a private high school
6	Unknown Public/Private	Unknown classification of high school as public or private
7	Is Parochial	Attended a parochial high school
8	Top 100 Private	Top 100 private school according to 2022 Niche Rankings
9	Top 100 Public	Top 100 public school according to 2022 U.S. News rankings
10	School Offers AP/SAT2 Fixed Effects	For each of the AP and SAT subject tests identified above, did at least one applicant in the high school-year report a score for it?
11	Rurality	Terms for U.S. Census Rurality Code
12	ZIP3 Fixed Effects	Terms for first three digits of high school zip code
13	State-year-basket Fixed Effects	Terms for each combination of Ivy-11 college applied to, high school state, and year of application, e.g., 'College X 2016 California'
14	Log State ACT Rank	Logarithm of the within state-year ranking of applicant's ACT score

Table S12. Variables included in Model 8, 'Location+HS'. Variables from Models 1 through 5 are also included, except for basket-year fixed effects, which are redundant with the included state-year-basket fixed effects.

Variant	Region	White base rate	Basket + year	SAT / ACT	GPA + AP + SAT2	ECs	Sex + Fam.	Early app	Legacy	Loc. + HS	All
Main model	E. Asian	12%	1.11	0.86	0.85	0.83	0.79	0.73	0.90	0.88	0.90
E. Asian and white	E. Asian	12%	1.12	0.87	0.84	0.81	0.78	0.73	0.89	0.88	0.89
Include recruits	E. Asian	13.8%	1.08	0.85	0.83	0.86	0.83	0.76	0.92	0.91	0.90
2015 only	E. Asian	12.8%	1.06	0.81	0.79	0.77	0.74	0.68	0.84	0.84	0.85
2016 only	E. Asian	12.4%	1.10	0.82	0.82	0.78	0.74	0.68	0.85	0.81	0.83
2017 only	E. Asian	11.9%	1.09	0.88	0.86	0.86	0.81	0.75	0.92	0.90	0.91
2018 only	E. Asian	11.2%	1.15	0.91	0.88	0.85	0.82	0.76	0.92	0.92	0.94
2019 only	E. Asian	11.9%	1.16	0.89	0.88	0.87	0.81	0.74	0.91	0.90	0.90
Northeast only	E. Asian	14.9%	1.18	0.92	0.87	0.85	0.80	0.72	0.92	0.86	0.86
California only	E. Asian	10.5%	0.89	0.66	0.81	0.80	0.77	0.76	0.86	0.90	0.95
Real ACT/GPA	E. Asian	11.5%	1.18	0.90	0.88	0.86	0.79	0.73	0.88	0.89	0.90
ACT $\geq$ 27	E. Asian	13.1%	1.12	0.86	0.84	0.82	0.76	0.71	0.86	0.87	0.89
Remove legacy	E. Asian	9.7%	1.30	1.00	0.96	0.94	0.90	0.82	0.91	0.99	0.90
US-educated parents	E. Asian	12.1%	0.98	0.86	0.87	0.85	0.80	0.76	0.86	0.88	0.86
Transcript senders	E. Asian	18.7%	1.36	1.06	0.99	0.95	0.89	0.82	1.00	0.92	0.93
Regular decision	E. Asian	7.6%	0.97	0.77	0.80	0.79	0.73	0.71	0.81	0.81	0.87
No transcript thres.	E. Asian	8.6%	1.19	0.90	0.87	0.85	0.82	0.76	0.92	0.90	0.91
20% transcript thres.	E. Asian	11.8%	1.12	0.87	0.85	0.84	0.80	0.74	0.90	0.89	0.90
0% transcript thres.	E. Asian	12.2%	1.11	0.86	0.84	0.83	0.79	0.72	0.89	0.88	0.89
Reweighted	E. Asian	12%	1.15	0.88	0.86	0.85	0.79	0.73	0.88	0.90	0.91
Leave one out max	E. Asian	12.1%	1.13	0.88	0.87	0.86	0.81	0.75	0.91	0.89	0.91
Leave one out min	E. Asian	10.9%	1.05	0.81	0.81	0.79	0.75	0.71	0.85	0.83	0.87

Table S13. Robustness checks of the main specification. Each variant of the main specification lists the corresponding value of the exponentiated East Asian coefficient for each of the nine models in the main analysis. Exponeniated coefficients are qualitatively similar across all specifications. **Detailed descriptions of each variant are on the next page.**

Detailed descriptions of each model variant:

- The ‘E. Asian and white’ variant fits the main specification only on East Asian and white applicants in the study pool, mimicking the effect of interacting race with each variable in the main model.
- The ‘Include recruits’ specification does not remove applicants who we believe may be recruited athletes.
- The ‘2015 only’ model fits the main model on only the 2015-2016 academic year application data, with a similar interpretation for the other variants whose names end in ‘only’.
- The ‘Real ACT/GPA’ model excludes applicants who do not report an ACT/SAT score and/or a high school GPA.
- The ‘ACT $\geq$ 27’ model removes applicants with an equivalent ACT below 27, as very few enrollees at Ivy-11 colleges have ACT scores below 27.
- The ‘Remove legacy’ model removes legacy applicants from the study pool, following a similar model choice in the Harvard v. SFFA court case.
- The ‘US-educated parents’ model excludes applicants whose parents exclusively attended undergraduate institutions outside of the United States.
- The ‘Transcript senders’ model includes only those applicants who sent a transcript to a specific college on the platform. These applicants have the strongest attendance signal, as the precision of our transcript-based enrollment heuristic is 97%.
- The ‘Regular decision’ model excludes applicants who applied early to only one college, sent a transcript to that college, and did not apply anywhere else. This is a likely signal of attendance at the school to which the student applied early.
- The ‘No transcript thres.’ allows all high school-years to be included in the analysis, and only excludes students with at least one application who sent the same number of transcripts as applications. The ‘20% transcript thres.’ model allows only applicants from high school-years for which less than 20% of applicants who submitted more than one application sent the same number of transcripts and applications. This model also removes all students with more than one application who sent the same number of transcripts as applications. The ‘0% transcript thres.’ model does not allow high-school years with any applicants who submitted more than one application and sent the same number of transcripts and applications.
- The ‘Reweighted’ model reweights the main model by the inverse likelihood that the given applicant attended a high school-year where no more than 5% of applicants who submitted more than one application also sent the same number of transcripts as applications. The corresponding propensity model is fit using the same covariates as Model 9, excluding the state-year-basket fixed effects.
- The ‘Leave one out’ variants assess the sensitivity of the Asian region coefficients to the set of 11 schools considered in the analysis. The exponentiated coefficients of the ‘Leave one out max’ and ‘Leave one out min’ variants are derived from fitting each model 11 times, where in each iteration we exclude applications from one of the 11 schools from the dataset. For each of the 9 model specifications, we report the maximum and minimum observed values of the exponentiated Asian region coefficient across 11 fitted models associated with each specification.

Variant	Region	White base rate	Basket + year	SAT / ACT	GPA + AP + SAT2	ECs	Sex + Fam.	Early app	Legacy	Loc. + HS	All
Main model	S. Asian	12%	0.66	0.56	0.59	0.51	0.51	0.52	0.61	0.60	0.70
S. Asian and white	S. Asian	12%	0.64	0.55	0.58	0.49	0.49	0.51	0.60	0.60	0.71
Include recruits	S. Asian	13.8%	0.62	0.53	0.55	0.52	0.52	0.53	0.61	0.61	0.69
2015 only	S. Asian	12.8%	0.63	0.53	0.57	0.50	0.49	0.50	0.59	0.59	0.69
2016 only	S. Asian	12.4%	0.65	0.53	0.57	0.49	0.48	0.50	0.58	0.56	0.67
2017 only	S. Asian	11.9%	0.67	0.58	0.62	0.53	0.53	0.54	0.63	0.62	0.71
2018 only	S. Asian	11.2%	0.65	0.56	0.59	0.51	0.51	0.52	0.61	0.59	0.68
2019 only	S. Asian	11.9%	0.69	0.58	0.62	0.53	0.52	0.52	0.61	0.61	0.68
Northeast only	S. Asian	14.9%	0.63	0.56	0.62	0.54	0.53	0.53	0.63	0.62	0.70
California only	S. Asian	10.5%	0.66	0.49	0.64	0.57	0.55	0.60	0.68	0.65	0.79
Real ACT/GPA	S. Asian	11.5%	0.70	0.58	0.61	0.53	0.51	0.53	0.60	0.61	0.69
ACT $\geq$ 27	S. Asian	13.1%	0.68	0.56	0.60	0.52	0.50	0.52	0.60	0.60	0.69
Remove legacy	S. Asian	9.7%	0.78	0.65	0.68	0.59	0.60	0.60	0.61	0.70	0.69
US-educated parents	S. Asian	12.1%	0.71	0.70	0.75	0.64	0.62	0.63	0.70	0.68	0.75
Transcript senders	S. Asian	18.7%	0.81	0.69	0.69	0.58	0.57	0.58	0.68	0.64	0.73
Regular decision	S. Asian	7.6%	0.60	0.52	0.58	0.53	0.51	0.52	0.60	0.59	0.68
No transcript thres.	S. Asian	8.6%	0.69	0.58	0.60	0.53	0.53	0.55	0.63	0.62	0.71
20% transcript thres.	S. Asian	11.8%	0.66	0.56	0.60	0.52	0.51	0.53	0.61	0.61	0.70
0% transcript thres.	S. Asian	12.2%	0.67	0.57	0.60	0.52	0.52	0.53	0.62	0.62	0.70
Reweighted	S. Asian	12%	0.67	0.56	0.59	0.52	0.51	0.53	0.60	0.62	0.71
Leave one out max	S. Asian	12.1%	0.67	0.57	0.60	0.52	0.51	0.53	0.62	0.61	0.71
Leave one out min	S Asian	10.9%	0.64	0.53	0.57	0.51	0.50	0.52	0.59	0.59	0.68

Table S14. Robustness checks of the main specification. Each variant of the main specification lists the corresponding value of the exponentiated South Asian coefficient for each of the nine models in the main analysis. Exponeniated coefficients are qualitatively similar across all specifications. Model variants are described in the caption of the corresponding figure for the exponentiated East Asian coefficient.

Variant	Region	White base rate	Basket + year	SAT / ACT	GPA + AP + SAT2	ECs	Sex + Fam.	Early app	Legacy	Loc. + HS	All
Main model	SE Asian	12%	0.64	0.73	0.78	0.83	0.81	0.84	0.88	0.94	1.02
SE Asian and white	SE Asian	12%	0.64	0.73	0.76	0.80	0.78	0.82	0.85	0.90	0.98
Include recruits	SE Asian	13.8%	0.60	0.69	0.72	0.81	0.80	0.83	0.87	0.92	0.99
2015 only	SE Asian	12.8%	0.64	0.76	0.79	0.83	0.82	0.88	0.90	1.01	1.15
2016 only	SE Asian	12.4%	0.62	0.71	0.75	0.82	0.78	0.79	0.86	0.91	0.98
2017 only	SE Asian	11.9%	0.64	0.74	0.79	0.84	0.83	0.85	0.91	0.97	1.04
2018 only	SE Asian	11.2%	0.64	0.72	0.77	0.82	0.80	0.85	0.87	0.94	1.06
2019 only	SE Asian	11.9%	0.67	0.75	0.80	0.84	0.79	0.81	0.85	0.92	0.99
Northeast only	SE Asian	14.9%	0.59	0.71	0.77	0.81	0.79	0.79	0.87	0.83	0.90
California only	SE Asian	10.5%	0.60	0.65	0.84	0.90	0.88	0.95	0.98	1.04	1.17
Real ACT/GPA	SE Asian	11.5%	0.69	0.81	0.85	0.88	0.82	0.85	0.88	0.96	1.03
ACT $\geq$ 27	SE Asian	13.1%	0.68	0.75	0.81	0.85	0.80	0.84	0.87	0.95	1.04
Remove legacy	SE Asian	9.7%	0.76	0.86	0.90	0.95	0.90	0.92	0.91	1.04	1.04
US-educated parents	SE Asian	12.1%	0.66	0.75	0.85	0.89	0.85	0.89	0.92	0.98	1.05
Transcript senders	SE Asian	18.7%	0.85	0.98	1.02	1.05	1.00	1.04	1.10	1.02	1.11
Regular decision	SE Asian	7.6%	0.70	0.79	0.86	0.91	0.84	0.85	0.91	0.97	1.05
No transcript thres.	SE Asian	8.6%	0.62	0.72	0.76	0.82	0.81	0.85	0.88	0.95	1.04
20% transcript thres.	SE Asian	11.8%	0.65	0.74	0.79	0.84	0.82	0.85	0.89	0.95	1.03
0% transcript thres.	SE Asian	12.2%	0.64	0.74	0.79	0.84	0.81	0.85	0.88	0.94	1.02
Reweighted	SE Asian	12%	0.71	0.83	0.88	0.94	0.88	0.91	0.94	1.05	1.13
Leave one out max	SE Asian	12.1%	0.66	0.76	0.82	0.88	0.84	0.88	0.92	0.95	1.05
Leave one out min	SE Asian	10.9%	0.58	0.64	0.68	0.72	0.69	0.75	0.75	0.82	0.90

Table S15. Robustness checks of the main specification. Each variant of the main specification lists the corresponding value of the exponentiated Southeast Asian coefficient for each of the nine models in the main analysis. Exponeniated coefficients are qualitatively similar across all specifications. Model variants are described in the caption of the corresponding figure for the exponentiated East Asian coefficient.

	Outcome: Inferred enrollment at Ivy-11 colleges with yield rates over 80%								
	Basket+year	SAT/ACT	GPA+AP+SAT2	Activities	Sex+Family	Early app	Legacy	Location+HS	All
	(1)	(2)	(3)	(4)	(5)	(6)	(7)	(8)	(9)
South Asian	0.70***	0.59***	0.68***	0.59***	0.57***	0.58***	0.71***	0.62***	0.75***
	(0.03)	(0.02)	(0.03)	(0.02)	(0.02)	(0.03)	(0.03)	(0.03)	(0.03)
Southeast Asian	0.81***	0.95	1.11	1.14*	1.10	1.14*	1.24***	1.14*	1.30***
	(0.04)	(0.05)	(0.06)	(0.06)	(0.06)	(0.07)	(0.07)	(0.07)	(0.08)
East Asian	1.09**	0.84***	0.83***	0.78***	0.74***	0.76***	0.87***	0.78***	0.91**
	(0.03)	(0.02)	(0.02)	(0.03)	(0.02)	(0.03)	(0.03)	(0.03)	(0.03)
Basket+year	X	X	X	X	X	X	X	X	X
SAT/ACT		X	X	X	X	X	X	X	X
GPA+AP+SAT2			X	X	X	X	X	X	X
Activities				X	X	X	X	X	X
Sex+Family					X	X	X	X	X
Early app						X			X
Legacy							X		X
Location+HS								X	X
Observations	136,743	136,743	136,743	136,743	136,743	136,743	136,743	136,743	136,743
In-sample AUC	0.63	0.72	0.78	0.8	0.81	0.83	0.82	0.83	0.85
Pseudo R-squared	0.03	0.08	0.13	0.16	0.16	0.2	0.19	0.19	0.25
White base rate	6.2%	6.2%	6.2%	6.2%	6.2%	6.2%	6.2%	6.2%	6.2%

*p<0.05; **p<0.01; ***p<0.001

Table S16. Replication of the models in Table 1 using only Ivy-11 schools with yield rates of at least 80% for the 2018–2019 application season (`https://www.usnews.com/education/best-colleges/articles/universities-colleges-where-students-are-eager-to-enroll`). To preserve anonymity, we do not reveal the number of included schools that meet this benchmark. The gaps in attendance rates among South and East Asian students relative to white students do not qualitatively differ from the corresponding gaps in the main results. However, the gaps between Southeast Asian students and white students increase substantially. For example, in the most saturated model, the observed gap grows from a statistically insignificant 2% higher odds for Southeast Asian students to a statistically significant 30% higher odds. One possible explanation for this difference is that Southeast Asian students received greater admissions boost from affirmative action policies at the schools with the highest yield rates relative to the Ivy-11 as a whole.

	Outcome: Enrolled at one of 37 selective schools								
	Basket+year	SAT/ACT	GPA+AP+SAT2	Activities	Sex+Family	Early app	Legacy	Location+HS	All
	(1)	(2)	(3)	(4)	(5)	(6)	(7)	(8)	(9)
South Asian	0.72^{***}	0.63^{***}	0.70^{***}	0.65^{***}	0.69^{***}	0.70^{***}	0.74^{***}	0.76^{***}	0.79^{***}
	(0.01)	(0.01)	(0.01)	(0.01)	(0.01)	(0.01)	(0.01)	(0.01)	(0.01)
Southeast Asian	0.70^{***}	0.78^{***}	0.84^{***}	0.88^{***}	0.90^{***}	0.93^{***}	0.94^{**}	0.98	1.02
	(0.01)	(0.01)	(0.02)	(0.02)	(0.02)	(0.02)	(0.02)	(0.02)	(0.02)
East Asian	1.13^{***}	0.94^{***}	0.98^{*}	0.98	1.01	0.94^{***}	1.06^{***}	1.08^{***}	1.03
	(0.01)	(0.01)	(0.01)	(0.01)	(0.01)	(0.01)	(0.01)	(0.01)	(0.01)
Basket+year	X	X	X	X	X	X	X	X	X
SAT/ACT		X	X	X	X	X	X	X	X
GPA+AP+SAT2			X	X	X	X	X	X	X
Activities				X	X	X	X	X	X
Sex+Family					X	X	X	X	X
Early app						X			X
Legacy							X		X
Location+HS								X	X
Observations	599,292	599,292	599,292	599,292	599,292	599,292	599,292	599,292	599,292
In-sample AUC	0.71	0.76	0.79	0.8	0.8	0.83	0.81	0.83	0.86
White base rate	19.6%	19.6%	19.6%	19.6%	19.6%	19.6%	19.6%	19.6%	19.6%

*p<0.05; **p<0.01; ***p<0.001

Table S17. Replication of the models in Table 1 using a larger group of schools. We include 27 of the 30 top national universities as ranked by U.S. News and World Report in 2019, in addition to the top 10 liberal arts colleges as ranked by U.S. News and World Report in the same year. The three excluded schools, UCLA, UC Berkeley, and Georgetown, do not accept applications via the postsecondary application platform. The gaps in attendance rates among Asian American students and white students are qualitatively similar to those in the main results, but the coefficient magnitudes are attenuated. For example, after adjusting for all observed application components, South Asian students have 21% lower odds of attending one of the 37 selective schools than similar white students. For the 11 Ivy-11 colleges in the main analysis, South Asian students have 30% lower odds. We note that the attendance gaps observed for the expanded set of schools are more weakly tied to admissions decisions, as the matriculation rates of the additional schools are substantially lower than those of the Ivy-11. However, that the same attendance gaps persist at a larger set of schools is concerning, and suggests that admissions policies of schools beyond the Ivy-11 might also exert disparate impacts on Asian American applicants.

	Outcome: Inferred enrollment at the Ivy-11								
	Basket+year	SAT/ACT	GPA+AP+SAT2	Activities	Sex+Family	Early app	Legacy	Location+HS	All
	(1)	(2)	(3)	(4)	(5)	(6)	(7)	(8)	(9)
White Female	0.89***	1.08***	1.03	1.01	1.01	1.05**	1.02	1.03	1.07***
	(0.01)	(0.02)	(0.02)	(0.02)	(0.02)	(0.02)	(0.02)	(0.02)	(0.02)
S. Asian Male	0.63***	0.53***	0.58***	0.51***	0.50***	0.50***	0.60***	0.58***	0.65***
	(0.02)	(0.01)	(0.02)	(0.02)	(0.02)	(0.02)	(0.02)	(0.02)	(0.02)
S. Asian Female	0.62***	0.63***	0.70***	0.58***	0.57***	0.61***	0.68***	0.67***	0.81***
	(0.02)	(0.02)	(0.02)	(0.02)	(0.02)	(0.02)	(0.02)	(0.02)	(0.03)
SE Asian Male	0.58***	0.65***	0.74***	0.81***	0.79***	0.83***	0.87**	0.91	0.99
	(0.03)	(0.03)	(0.04)	(0.04)	(0.04)	(0.04)	(0.04)	(0.05)	(0.05)
SE Asian Female	0.61***	0.85***	0.93	0.93	0.89*	0.96	0.98	1.04	1.16**
	(0.03)	(0.04)	(0.04)	(0.04)	(0.04)	(0.05)	(0.05)	(0.05)	(0.06)
E. Asian Male	0.96	0.76***	0.75***	0.74***	0.70***	0.65***	0.81***	0.79***	0.80***
	(0.02)	(0.02)	(0.02)	(0.02)	(0.02)	(0.02)	(0.02)	(0.02)	(0.02)
E. Asian Female	1.12***	1.02	1.00	0.93**	0.89***	0.85***	1.02	1.01	1.06*
	(0.02)	(0.02)	(0.02)	(0.02)	(0.02)	(0.02)	(0.02)	(0.03)	(0.03)
Basket+year	X	X	X	X	X	X	X	X	X
SAT/ACT		X	X	X	X	X	X	X	X
GPA+AP+SAT2			X	X	X	X	X	X	X
Activities				X	X	X	X	X	X
Sex+Family					X	X	X	X	X
Early app						X			X
Legacy							X		X
Location+HS								X	X
Observations	292,795	292,795	292,795	292,795	292,795	292,795	292,795	292,795	292,795
In-sample AUC	0.66	0.75	0.79	0.82	0.82	0.86	0.83	0.84	0.88
Pseudo R-squared	0.06	0.12	0.17	0.21	0.21	0.27	0.23	0.25	0.32
White base rate	12.8%	12.8%	12.8%	12.8%	12.8%	12.8%	12.8%	12.8%	12.8%

*p<0.05; **p<0.01; ***p<0.001

Table S18. Replication of the models in Table 1 when interacting ethnicity and sex. White male applicants are the base category. Across ethnicities, applicants who identify as female attend an Ivy-11 school at significantly higher rates than male applicants of the same ethnicity with similar application profiles. South Asian and East Asian males have significantly lower odds of attendance than white males with similar application factors, with a larger gap for South Asian males. South Asian females are the only group of female applicants who have lower odds of attendance than white males with similar qualifications. These results complement prior work that has shown that Asian American male applicants may face a larger admissions penalty relative to Asian American female applicants at elite colleges [Arcidiacono et al., 2022].

	Outcome: Inferred enrollment at one of 11 Ivy Plus colleges								
	Basket+year	SAT/ACT	GPA+AP+SAT2	Activities	Sex+Family	Early app	Legacy	Location+HS	All
	(1)	(2)	(3)	(4)	(5)	(6)	(7)	(8)	(9)
White+Asian	1.01	0.92**	0.90***	0.93**	0.91**	0.89***	0.90***	0.96	0.92**
	(0.03)	(0.02)	(0.03)	(0.03)	(0.03)	(0.03)	(0.03)	(0.03)	(0.03)
Asian	0.86***	0.71***	0.77***	0.70***	0.67***	0.65***	0.80***	0.76***	0.83***
	(0.01)	(0.01)	(0.01)	(0.01)	(0.01)	(0.01)	(0.01)	(0.01)	(0.02)
Basket+year	X	X	X	X	X	X	X	X	X
SAT/ACT		X	X	X	X	X	X	X	X
GPA+AP+SAT2			X	X	X	X	X	X	X
Activities				X	X	X	X	X	X
Sex+Family					X	X	X	X	X
Early app						X			X
Legacy							X		X
Location+HS								X	X
Observations	292,795	292,795	292,795	292,795	292,795	292,795	292,795	292,795	292,795
In-sample AUC	0.65	0.75	0.79	0.81	0.82	0.86	0.83	0.84	0.88
Pseudo R-squared	0.05	0.12	0.17	0.2	0.21	0.27	0.23	0.25	0.32
White base rate	12%	12%	12%	12%	12%	12%	12%	12%	12%

*p<0.05; **p<0.01; ***p<0.001

Table S19. Replication of the models in Table 1 after disaggregating Asian American applicants into those who identify solely as Asian American, and applicants who identify as both Asian American and white. Among Asian American applicants, 86% identify solely as Asian American, with 14% identifying as both Asian American and white. Of applicants who identify as both Asian American and white, 57% identify as East Asian, 17% identify as South Asian, and 26% identify as Southeast Asian. Across most specifications, applicants who identify as both Asian American and white are significantly less likely than similarly qualified white applicants to attend an Ivy-11 school. However, effect sizes are substantially attenuated relative to applicants who identify solely as Asian American.

Race	ZIP 1	ACT	Non-legacy applicants	Non-legacy enrollees	Legacy applicants	Legacy enrollees
White	0	32	3,321	199	411	102
White	0	33	4,652	409	705	166
White	0	34	5,201	746	942	357
White	0	35	4,380	1,058	980	449
White	0	36	1,600	609	451	306
White	1	32	3,236	286	285	77
White	1	33	4,455	516	622	214
White	1	34	5,023	1,002	849	373
White	1	35	4,093	1,198	898	538
White	1	36	1,374	555	404	295
White	2	32	1,618	77	155	21
White	2	33	2,408	159	252	33
White	2	34	2,770	285	385	119
White	2	35	2,298	461	410	177
White	2	36	868	252	184	108
White	3	32	1,148	49	59	14
White	3	33	1,630	111	103	23
White	3	34	1,949	207	157	47
White	3	35	1,717	253	151	74
White	3	36	708	191	78	45
White	4	32	1,022	37		
White	4	33	1,400	78	77	13
White	4	34	1,521	128	82	29
White	4	35	1,364	185	81	32
White	4	36	464	78		
White	5	32	548	22		
White	5	33	768	59		
White	5	34	821	55	54	16
White	5	35	745	111	65	31
White	5	36	270	64		
White	6	32	1,082	52	65	17
White	6	33	1,606	100	95	19
White	6	34	1,890	193	156	52
White	6	35	1,729	258	135	43
White	6	36	716	132	75	42
White	7	32	692	31		
White	7	33	924	36		
White	7	34	1,263	106	78	21
White	7	35	1,143	148	85	35
White	7	36	494	105	54	31
White	8	32	786	36	59	8
White	8	33	1,052	72	83	19
White	8	34	1,148	117	101	36
White	8	35	995	152	91	34
White	8	36	334	76	53	29
White	9	32	2,418	107	285	47
White	9	33	3,396	195	431	88
White	9	34	3,969	416	583	175
White	9	35	3,298	530	584	217
White	9	36	1,108	276	243	127

Table S20. Aggregated counts of white applicants and enrollees across groups defined by geography, equivalent ACT score, and legacy status. Attendance is proxied by observing whether a final transcript is sent to an Ivy-11 college. “ZIP 1” refers to the first digit of the student’s high school ZIP code. To preserve confidentiality, legacy and non-legacy applicant cell counts with fewer than 50 applicants are redacted, along with the corresponding count of enrollees. Further, legacy and non-legacy enrollee cell counts of 0 are redacted, along with the corresponding count of applicants.

Ethnicity	ZIP 1	ACT	Non-legacy applicants	Non-legacy enrollees	Legacy applicants	Legacy enrollees
Asian	0	32	1,109	56	51	12
Asian	0	33	1,988	146	86	20
Asian	0	34	3,050	353	181	50
Asian	0	35	4,037	948	209	104
Asian	0	36	2,538	1,027	86	56
Asian	1	32	1,596	104		
Asian	1	33	2,363	265	73	27
Asian	1	34	3,126	487	115	55
Asian	1	35	3,204	985	165	85
Asian	1	36	1,569	733	90	63
Asian	2	32	628	19		
Asian	2	33	1,067	44		
Asian	2	34	1,513	154	51	8
Asian	2	35	1,996	432	97	51
Asian	2	36	1,103	358		
Asian	3	32	432	9		
Asian	3	33	754	41		
Asian	3	34	1,147	95		
Asian	3	35	1,347	227		
Asian	3	36	887	297		
Asian	4	32	269	10		
Asian	4	33	434	23		
Asian	4	34	730	72		
Asian	4	35	915	172		
Asian	4	36	604	162		
Asian	5	32	143	7		
Asian	5	33	193	15		
Asian	5	34	288	30		
Asian	5	35	375	73		
Asian	5	36	233	85		
Asian	6	32	412	10		
Asian	6	33	616	33		
Asian	6	34	876	77		
Asian	6	35	1,125	162		
Asian	6	36	739	231		
Asian	7	32	321	9		
Asian	7	33	604	27		
Asian	7	34	987	80		
Asian	7	35	1,529	213		
Asian	7	36	1,040	250		
Asian	8	32	222	10		
Asian	8	33	327	21		
Asian	8	34	512	58		
Asian	8	35	553	128		
Asian	8	36	344	122		
Asian	9	32	2,100	70	68	4
Asian	9	33	3,473	163	126	25
Asian	9	34	5,348	379	205	56
Asian	9	35	6,692	877	255	83
Asian	9	36	3,523	771	132	59

Table S21. Aggregated counts of Asian American applicants and enrollees across groups defined by geography, equivalent ACT score, and legacy status. Attendance is proxied by observing whether a final transcript is sent to an Ivy-11 college. "ZIP 1" refers to the first digit of the student's high school ZIP code. To preserve confidentiality, legacy and non-legacy applicant cell counts with fewer than 50 applicants are redacted, along with the corresponding count of enrollees. Further, legacy and non-legacy enrollee cell counts of 0 are redacted, along with the corresponding count of applicants.

Ethnicity	ZIP 1	ACT	Non-legacy applicants	Non-legacy enrollees	Legacy applicants	Legacy enrollees
South Asian	0	32	422	8		
South Asian	0	33	883	42		
South Asian	0	34	1,314	106		
South Asian	0	35	1,561	277		
South Asian	0	36	894	285		
South Asian	1	32	516	28		
South Asian	1	33	737	65		
South Asian	1	34	968	148		
South Asian	1	35	928	244		
South Asian	1	36	366	155		
South Asian	2	32	264	4		
South Asian	2	33	460	21		
South Asian	2	34	615	47		
South Asian	2	35	808	135		
South Asian	2	36	372	100		
South Asian	3	32	185	4		
South Asian	3	33	363	14		
South Asian	3	34	551	32		
South Asian	3	35	594	86		
South Asian	3	36	365	101		
South Asian	4	32	128	3		
South Asian	4	33	198	8		
South Asian	4	34	352	33		
South Asian	4	35	424	70		
South Asian	4	36	240	48		
South Asian	5	32	55	3		
South Asian	5	33	79	3		
South Asian	5	34	117	12		
South Asian	5	35	152	28		
South Asian	5	36	60	17		
South Asian	6	32	166	4		
South Asian	6	33	260	9		
South Asian	6	34	387	25		
South Asian	6	35	483	54		
South Asian	6	36	272	66		
South Asian	7	32	142	4		
South Asian	7	33	287	11		
South Asian	7	34	503	32		
South Asian	7	35	707	84		
South Asian	7	36	444	74		
South Asian	8	32	60	3		
South Asian	8	33	110	4		
South Asian	8	34	172	16		
South Asian	8	35	196	40		
South Asian	8	36	132	45		
South Asian	9	32	434	16		
South Asian	9	33	781	24		
South Asian	9	34	1,232	61		
South Asian	9	35	1,791	159		
South Asian	9	36	997	195		

Table S22. Aggregated counts of South Asian applicants and enrollees across groups defined by geography, equivalent ACT score, and legacy status. Attendance is proxied by observing whether a final transcript is sent to an Ivy-11 college. “ZIP 1” refers to the first digit of the student’s high school ZIP code. To preserve confidentiality, legacy and non-legacy applicant cell counts with fewer than 50 applicants are redacted, along with the corresponding count of enrollees. Further, legacy and non-legacy enrollee cell counts of 0 are redacted, along with the corresponding count of applicants.

Ethnicity	ZIP 1	ACT	Non-legacy applicants	Non-legacy enrollees	Legacy applicants	Legacy enrollees
Southeast Asian	0	32	189	7		
Southeast Asian	0	33	253	21		
Southeast Asian	0	34	270	37		
Southeast Asian	0	35	263	69		
Southeast Asian	0	36	82	30		
Southeast Asian	1	32	208	9		
Southeast Asian	1	33	298	45		
Southeast Asian	1	34	293	41		
Southeast Asian	1	35	217	53		
Southeast Asian	1	36	92	38		
Southeast Asian	2	32	105	5		
Southeast Asian	2	33	145	3		
Southeast Asian	2	34	157	15		
Southeast Asian	2	35	135	24		
Southeast Asian	2	36	52	16		
Southeast Asian	3	32	78	2		
Southeast Asian	3	33	112	11		
Southeast Asian	3	34	128	16		
Southeast Asian	3	35	115	23		
Southeast Asian	3	36				
Southeast Asian	4	32				
Southeast Asian	4	33	57	4		
Southeast Asian	4	34	52	4		
Southeast Asian	4	35	57	12		
Southeast Asian	4	36				
Southeast Asian	5	32				
Southeast Asian	5	33				
Southeast Asian	5	34				
Southeast Asian	5	35				
Southeast Asian	5	36				
Southeast Asian	6	32	71	3		
Southeast Asian	6	33	108	7		
Southeast Asian	6	34	98	9		
Southeast Asian	6	35	80	9		
Southeast Asian	6	36				
Southeast Asian	7	32	70	2		
Southeast Asian	7	33	117	5		
Southeast Asian	7	34	109	9		
Southeast Asian	7	35	171	21		
Southeast Asian	7	36	88	18		
Southeast Asian	8	32	52	3		
Southeast Asian	8	33	53	5		
Southeast Asian	8	34	68	11		
Southeast Asian	8	35	58	11		
Southeast Asian	8	36				
Southeast Asian	9	32	470	17		
Southeast Asian	9	33	648	38		
Southeast Asian	9	34	814	56		
Southeast Asian	9	35	771	119		
Southeast Asian	9	36	249	55		

Table S23. Aggregated counts of Southeast Asian applicants and enrollees across groups defined by geography, equivalent ACT score, and legacy status. Attendance is proxied by observing whether a final transcript is sent to an Ivy-11 college. "ZIP 1" refers to the first digit of the student's high school ZIP code. To preserve confidentiality, legacy and non-legacy applicant cell counts with fewer than 50 applicants are redacted, along with the corresponding count of enrollees. Further, legacy and non-legacy enrollee cell counts of 0 are redacted, along with the corresponding count of applicants.

Ethnicity	ZIP 1	ACT	Non-legacy applicants	Non-legacy enrollees	Legacy applicants	Legacy enrollees
East Asian	0	32	498	41		
East Asian	0	33	852	83	61	18
East Asian	0	34	1,466	210	124	34
East Asian	0	35	2,213	602	156	81
East Asian	0	36	1,562	712	65	47
East Asian	1	32	872	67		
East Asian	1	33	1,328	155	57	23
East Asian	1	34	1,865	298	82	37
East Asian	1	35	2,059	688	111	57
East Asian	1	36	1,111	540	62	46
East Asian	2	32	259	10		
East Asian	2	33	462	20		
East Asian	2	34	741	92		
East Asian	2	35	1,053	273	62	33
East Asian	2	36	679	242		
East Asian	3	32	169	3		
East Asian	3	33	279	16		
East Asian	3	34	468	47		
East Asian	3	35	638	118		
East Asian	3	36	480	182		
East Asian	4	32	103	3		
East Asian	4	33	179	11		
East Asian	4	34	326	35		
East Asian	4	35	434	90		
East Asian	4	36	348	109		
East Asian	5	32	66	2		
East Asian	5	33	80	7		
East Asian	5	34	131	13		
East Asian	5	35	186	41		
East Asian	5	36	156	62		
East Asian	6	32	175	3		
East Asian	6	33	248	17		
East Asian	6	34	391	43		
East Asian	6	35	562	99		
East Asian	6	36	435	153		
East Asian	7	32	109	3		
East Asian	7	33	200	11		
East Asian	7	34	375	39		
East Asian	7	35	651	108		
East Asian	7	36	508	158		
East Asian	8	32	110	4		
East Asian	8	33	164	12		
East Asian	8	34	272	31		
East Asian	8	35	299	77		
East Asian	8	36	181	73		
East Asian	9	32	1,196	37	53	3
East Asian	9	33	2,044	101	92	19
East Asian	9	34	3,302	262	163	45
East Asian	9	35	4,130	599	198	65
East Asian	9	36	2,277	521	100	43

Table S24. Aggregated counts of East Asian applicants and enrollees across groups defined by geography, equivalent ACT score, and legacy status. Attendance is proxied by observing whether a final transcript is sent to an Ivy-11 college. "ZIP 1" refers to the first digit of the student's high school ZIP code. To preserve confidentiality, legacy and non-legacy applicant cell counts with fewer than 50 applicants are redacted, along with the corresponding count of enrollees. Further, legacy and non-legacy enrollee cell counts of 0 are redacted, along with the corresponding count of applicants.

Ethnicity	ACT	Non-legacy applicants	Non-legacy enrollees	Legacy applicants	Legacy enrollees
White	32	15,871	896	1,415	294
White	33	22,291	1,735	2,459	591
White	34	25,555	3,255	3,387	1,225
White	35	21,762	4,354	3,480	1,630
White	36	7,936	2,338	1,613	1,021
South Asian	32	2,372	77		
South Asian	33	4,158	201	70	11
South Asian	34	6,211	512	123	42
South Asian	35	7,644	1,177	159	78
South Asian	36	4,142	1,086	73	43
Southeast Asian	32	1,303	54		
Southeast Asian	33	1,825	144	51	7
Southeast Asian	34	2,029	203	75	15
Southeast Asian	35	1,904	345	81	32
Southeast Asian	36	701	198		
East Asian	32	3,557	173	168	22
East Asian	33	5,836	433	268	69
East Asian	34	9,337	1,070	467	146
East Asian	35	12,225	2,695	599	263
East Asian	36	7,737	2,752	301	185

Table S25. Aggregated counts of legacy and non-legacy applicants and enrollees across ethnicity and ACT groups. This data can be used to replicate Figure 1, and the bottom panel of Figure 2. Attendance is proxied by observing whether a final transcript is sent to an Ivy-11 college. To preserve confidentiality, legacy and non-legacy applicant cell counts with fewer than 50 applicants are redacted, along with the corresponding enrollee count. Further, legacy and non-legacy enrollee cell counts of 0 are redacted, along with the corresponding count of applicants.

Race	ACT	Non-legacy applicants	Non-legacy enrollees	Legacy applicants	Legacy enrollees
Asian	32	7,232	304	231	36
Asian	33	11,819	778	389	87
Asian	34	17,577	1,785	665	203
Asian	35	21,773	4,217	839	373
Asian	36	12,580	4,036	409	242
White	32	15,871	896	1,415	294
White	33	22,291	1,735	2,459	591
White	34	25,555	3,255	3,387	1,225
White	35	21,762	4,354	3,480	1,630
White	36	7,936	2,338	1,613	1,021

Table S26. Aggregated counts of legacy and non-legacy applicants and enrollees across ACT and race groups. This data can be used to replicate the upper panel of Figure 2. Attendance is proxied by observing whether a final transcript is sent to an Ivy-11 college. To preserve confidentiality, legacy and non-legacy applicant cell counts with fewer than 50 applicants are redacted, along with the corresponding enrollee count. Further, legacy and non-legacy enrollee cell counts of 0 are redacted, along with the corresponding count of applicants.

State	Abbv.	Prop. Asian	Prop. Attend	Mean ACT
Alabama	AL	0.38	0.10	33.1
Alaska	AK	0.15	0.22	31.5
Arizona	AZ	0.41	0.12	32.8
Arkansas	AR	0.36	0.09	32.6
California	CA	0.58	0.11	32.7
Colorado	CO	0.25	0.09	32.2
Connecticut	CT	0.26	0.17	32.1
Delaware	DE	0.40	0.14	31.5
District of Columbia	DC	0.17	0.26	33.3
Florida	FL	0.31	0.13	32.4
Georgia	GA	0.49	0.09	33.1
Hawaii	HI	0.85	0.17	31.6
Idaho	ID	0.22	0.08	32.1
Illinois	IL	0.35	0.11	32.3
Indiana	IN	0.25	0.06	31.8
Iowa	IA	0.33	0.08	33.0
Kansas	KS	0.38	0.09	33.1
Kentucky	KY	0.18	0.12	32.6
Louisiana	LA	0.26	0.11	32.8
Maine	ME	0.13	0.19	30.9
Maryland	MD	0.41	0.12	32.8
Massachusetts	MA	0.32	0.16	32.4
Michigan	MI	0.41	0.08	32.6
Minnesota	MN	0.29	0.10	32.6
Mississippi	MS	0.33	0.10	33.2
Missouri	MO	0.29	0.10	32.8
Montana	MT	0.08	0.20	31.9
Nebraska	NE	0.31	0.12	32.7
Nevada	NV	0.44	0.10	32.3
New Hampshire	NH	0.28	0.17	31.8
New Jersey	NJ	0.49	0.14	32.6
New Mexico	NM	0.31	0.14	32.3
New York	NY	0.39	0.22	32.1
North Carolina	NC	0.33	0.12	32.3
North Dakota	ND	0.26	0.27	32.5
Ohio	OH	0.31	0.09	32.6
Oklahoma	OK	0.42	0.06	32.4
Oregon	OR	0.39	0.08	32.2
Pennsylvania	PA	0.31	0.15	31.7
Rhode Island	RI	0.20	0.22	31.0
South Carolina	SC	0.24	0.12	32.3
South Dakota	SD	0.24	0.21	32.3
Tennessee	TN	0.29	0.11	32.9
Texas	TX	0.52	0.10	33.3
Utah	UT	0.21	0.15	32.3
Vermont	VT	0.11	0.19	30.8
Virginia	VA	0.41	0.10	32.6
Washington	WA	0.51	0.07	32.7
West Virginia	WV	0.31	0.20	32.1
Wisconsin	WI	0.23	0.09	32.5
Wyoming	WY	0.07	0.14	31.7

Table S27. Data used to construct Figures 3, S1, and S2.

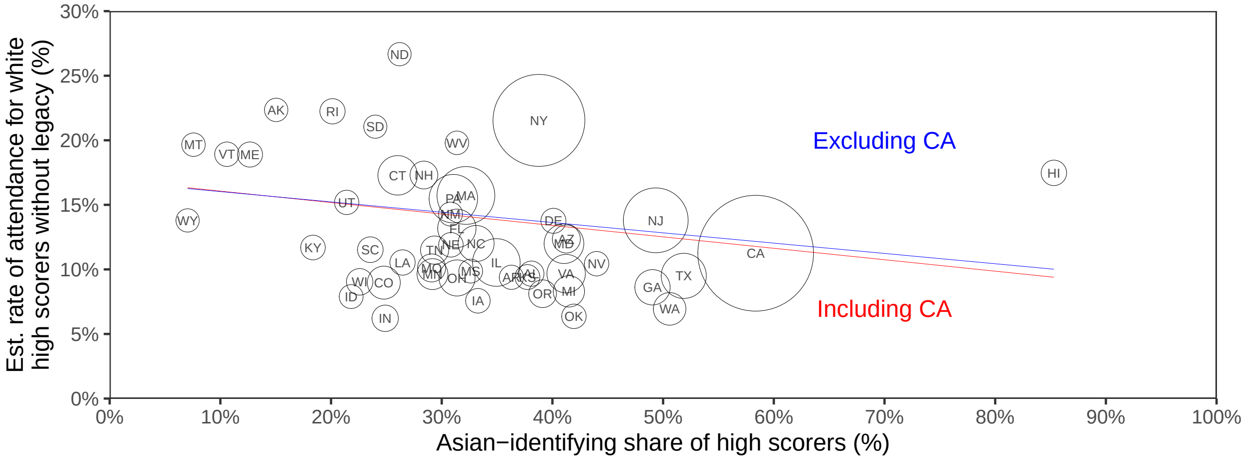

Figure S1. For each U.S. state, estimated rate of attendance at any one of the Ivy-11 colleges for non-legacy white applicants with an ACT-equivalent score at or above 32, with the proportion of high-scoring white and Asian applicants who identify as Asian on the horizontal axis. We report attendance rates of non-legacy white applicants to better isolate the impact of geography on attendance from the potential impacts of legacy status and race itself. Larger point sizes indicate a higher number of high-scoring white and Asian applicants from the state. The red least-squares regression line is weighted by the same count of high-scoring white and Asian American applicants from each state. The blue line excludes applicants from California. States with a greater share of Asian American applicants have, on average, lower attendance rates for high-scoring and non-legacy white applicants. This pattern holds even if applicants from California are excluded. Table S27 displays the data used to construct this plot.

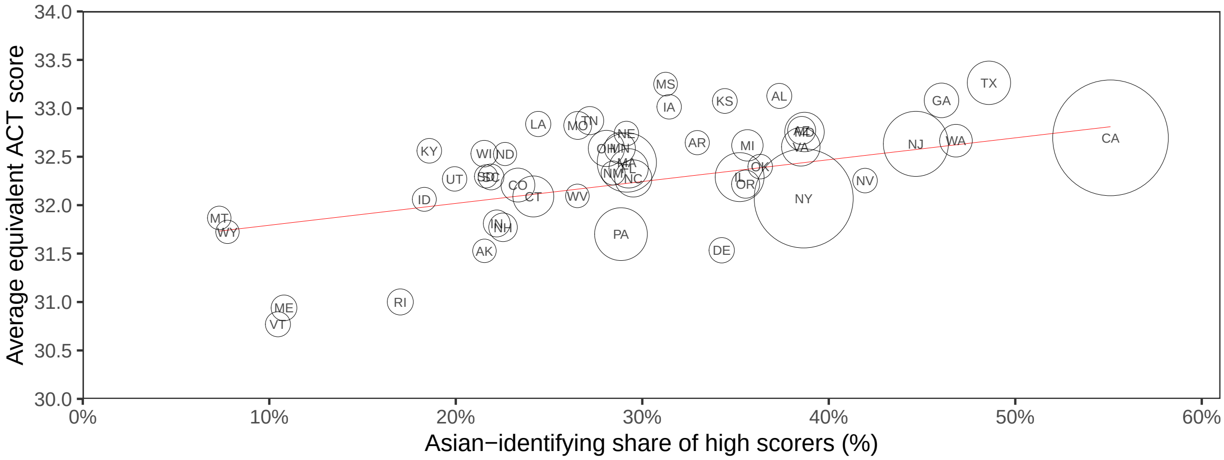

Figure S2. For each U.S. state, mean equivalent ACT score among applicants who reported an ACT score, with the proportion of high-scoring white and Asian applicants who identify as Asian on the horizontal axis. Hawaii is excluded from the plot due to its exceptionally high share of Asian American applicants. Hawaii's mean equivalent ACT score is 31.6. Larger point sizes indicate a higher number of high-scoring white and Asian applicants from the state. The red least-squares regression line is weighted by the same count of white and Asian American applicants from each state. Table S27 displays the data used to construct this plot.

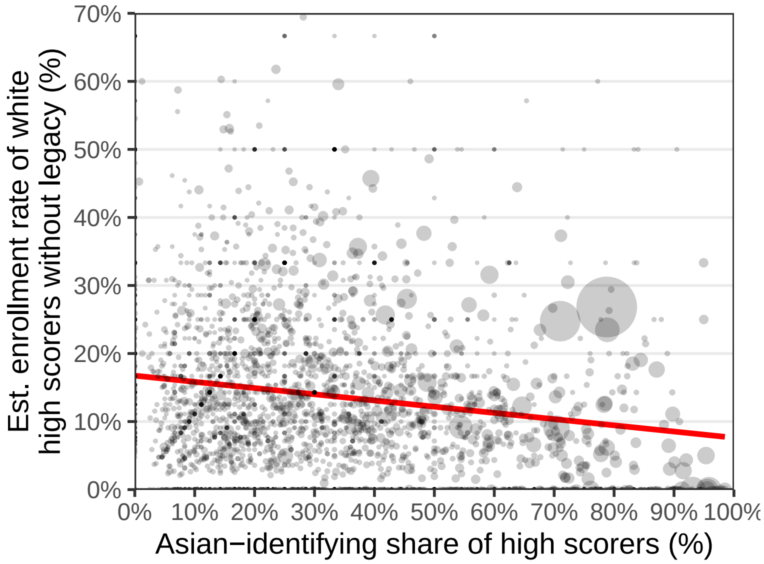

Figure S3. For each high school in the study pool, rate of attendance at any Ivy-11 college for non-legacy white applicants with an ACT-equivalent score at or above 32, with the proportion of high-scoring white and Asian American applicants who identify as Asian American on the horizontal axis. We report attendance rates of non-legacy white applicants to better isolate the impact of geography on attendance from the potential impacts of legacy status and race itself. Point sizes are proportional to the number of high-scoring white applicants and Asian applicants to the considered institutions who attend the given high school. The red least-squares regression line is weighted by the same count of high-scoring white and Asian American applicants from the given high school. High schools with a greater share of Asian American applicants have, on average, lower attendance rates for high-scoring non-legacy white applicants.

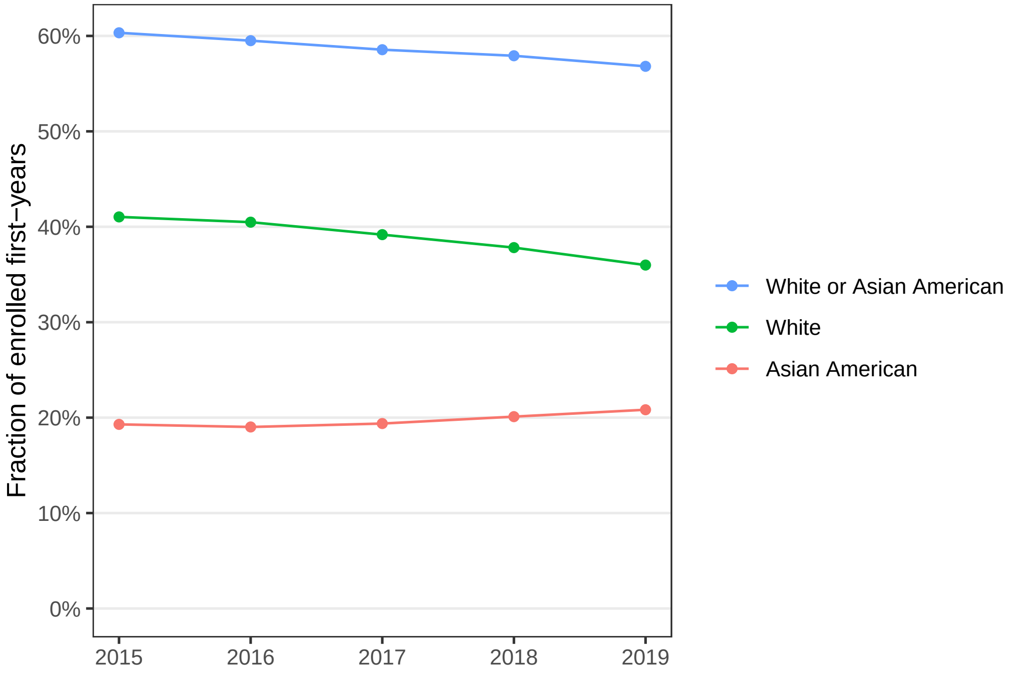

Figure S4. Fraction of observed enrollments at Ivy-11 colleges attributed to Asian American and white enrollees. The overall share of enrollments attributed to Asian American and white enrollees decreases slightly over the five years included in the analysis. The hypothetical policies described in the main analysis assume that this share remains approximately constant regardless of application year.

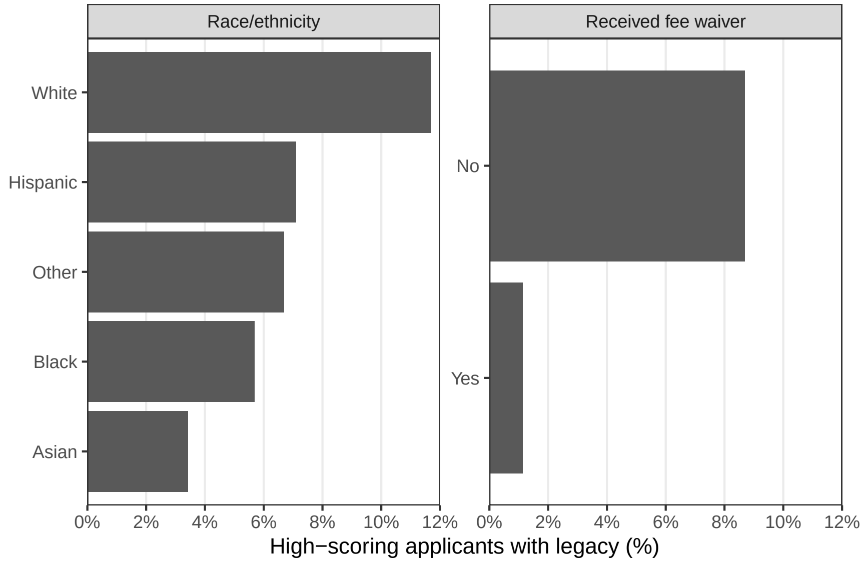

Figure S5. The proportion of applicants with one or more parents who attended an Ivy-11 college as an undergraduate, by race/ethnicity and fee waiver status. The pool of applicants in this figure is the same as the main analysis, but does not apply the filters for race or ethnicity. Following the convention of related work, we denote both Black Hispanic and Black non-Hispanic applicants with the label "Black", with the "Hispanic" label referring to white Hispanic applicants and the "white" label referring to white non-Hispanic applicants. White applicants are the most likely to have legacy status, and Asian American applicants are the least likely to have legacy status.

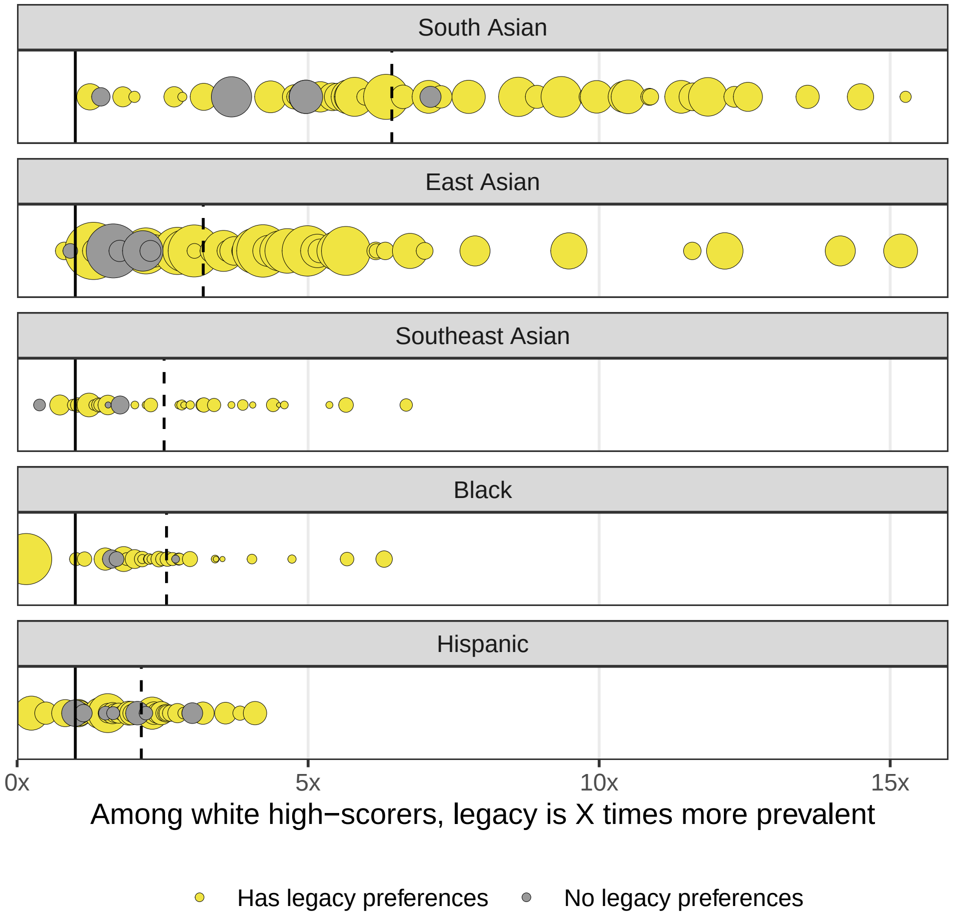

Figure S6. For a large set of selective colleges and universities, ratio of the prevalence of high-scoring white applicants with legacy status divided by the corresponding proportion for applicants who identify with other race and ethnic groups. For example, if 10% of white applicants to a particular school have legacy status, compared to 1% of South Asian applicants, then the ratio for South Asian applicants to that school is 10. The included schools are a combination of selective universities and colleges for which we have application materials, including private universities, public flagship universities, and liberal arts colleges. We define "high-scoring" as having an equivalent ACT score at least as high as the median equivalent ACT score of enrollees. Point sizes are approximately proportional to the number of applications from students of each race and ethnicity. The yellow points indicate schools with publicly-stated legacy preferences in admissions, while the gray points indicate schools that have stated publicly that they do not consider legacy in admissions. Schools with unknown legacy preferences are excluded. Schools with less than 1,000 applicants from the given race or ethnic group are hidden. High-scoring white applicants are, on average, approximately six to seven times more likely to have legacy status than high-scoring South Asian applicants, and two to three times more likely than high-scoring East Asian, Southeast Asian, Black, and Hispanic applicants. These results suggest that legacy preferences may disproportionately benefit white applicants at schools across the country. We note, however, that there is substantial heterogeneity in school-level legacy ratios, so the extent of the benefit likely differs across campuses.

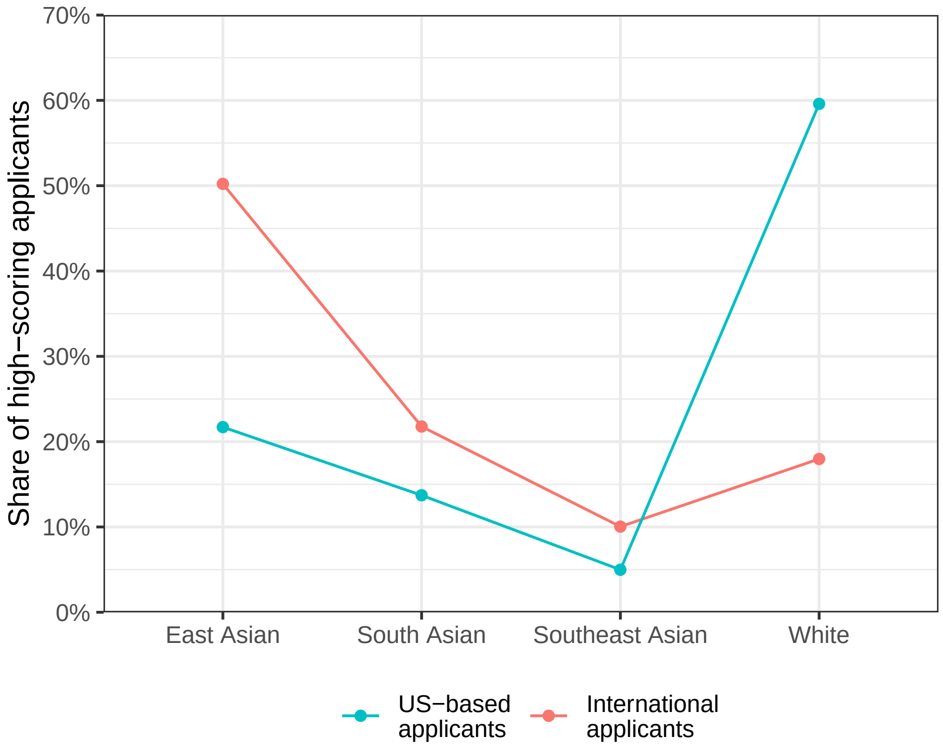

Figure S7. Ethnicity of high-scoring white- and Asian-identifying applicants, disaggregated by international applicant status. Applicants from East, South, and Southeast Asia make up a larger share of the international applicant pool than the U.S.-based applicant pool. There is an especially pronounced difference for East Asian applicants. This difference may, in part, drive the observed differences in attendance rates between similarly-qualified white and Asian applicants from U.S. high schools.

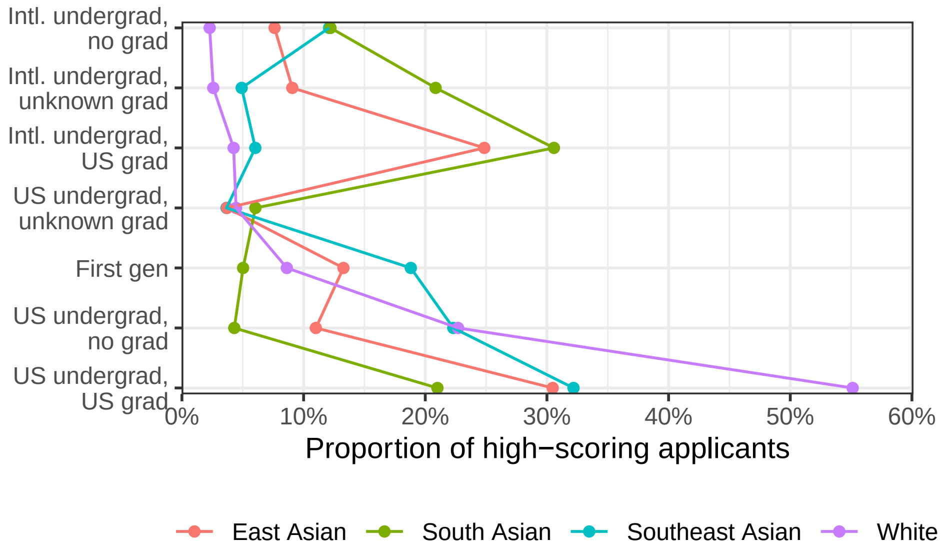

Figure S8. Educational background of the parents of high-scoring U.S.-based applicants identifying as white or Asian. East and Southeast Asian applicants are far more likely than white applicants to have parents with undergraduate degrees from an international institution. This difference may, in part, be responsible for observed differences in attendance rates for similarly qualified Asian and white applicants. For example, attending a U.S.-based college may provide additional knowledge of how to best frame a college application for admission to a selective institution. If one parent does not hold an undergraduate degree, we only consider the undergraduate education of the other parent. The same is true for graduate degrees. We can detect whether a parent attended graduate school located in the United States if and only if the institution also has an undergraduate program. "Unknown grad" implies that the parent attended either an international graduate school, or a U.S.-based graduate institution without an undergraduate program.
